# Electrochemical Determination of Bisphenol a Using a Drop‐Dry Modified Gold Electrode with Metal–Organic Framework, Quantum Dots, and their Composite

**DOI:** 10.1002/open.202500327

**Published:** 2025-08-07

**Authors:** Solomon O. Oloyede, Peter A. Ajibade

**Affiliations:** ^1^ School of chemistry and physics University of Kwazulu‐Natal Private Bag X01 Pietermaritzburg 3209 South Africa

**Keywords:** bisphenol A, composites, electrochemistry, metal‐organic framework, quantum dots

## Abstract

A modified gold electrode with metal‐organic frameworks (MOFs), quantum dots (QTs) and their composite are fabricated to determine bisphenol A. The chemically modified sensors are characterized using ultraviolet‐visible, Fourier transform infrared spectroscopy spectra, X‐ray diffraction, scanning electron microscopy, and ransmission electron microscopy. Upon examining the electrochemical characteristics of the fabricated sensors, it is discovered that the QDs@MOFs conjugate performs better than the metal‐organic frameworks and quantum dots, which could be attributed to the better conductivity of the conjugate. The effects of pH, accumulation time, and sensor concentration are studied at optimal condition. Over a wide range of bisphenol A (BPA) concentrations (1 μM–14 μM), the limit of detection is found to be 0.470 μM and the limit of quantitation is 1.425 μM. The results indicate that the electrochemical sensor fabricated from composite modified gold electrode is efficient for the detection of bisphenol A. The stability and reproducibility of the sensor are also evaluated.

## Graphical abstract

Metal‐organic frameworks, quantum dots and their composite are used as electrochemical sensors at the surface of a gold electrode for bisphenol A (BPA) determination. Electrochemical response of the composite modified gold electrode shows excellence performance with a very low detection limit. The stability, reproducibility, and selectivity properties of the composite‐modified electrode have been confirmed and ascertained. Real water sample analysis indicates that the fabricated composite modified electrode could be used for practical applications.

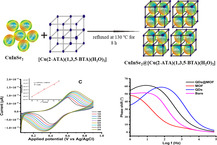

## 1 Introduction

Over the past decades, there has been rapid increase in the release of toxic pollutants into the ecosystem because of rapid urbanization and industrialization.^[^ ^1^ ^,^ ^2^ ^]^ The manufacturing of huge amount of biomedical and synthetic chemicals have resulted in the discharge of unwanted pollutants, in the soil, water, air, and food.^[^ ^3^ ^,^ ^4^ ^]^ Among these pollutants, endocrine‐disrupting chemicals (EDCs) emerge as ones of the more harmful and dangerous exogenous agents.^[^ ^5^ ^]^ Phthalates, polychlorinated biphenyls (PCBs), dioxins, fungicides, pesticides, methoxychlor, chlorpyrifos, phytoestrogens, pharmaceutical agents, and bisphenols (BPs) are the most widespread endocrine disruptors.^[^ ^6^ ^–^ ^7^ ^,^ ^8^ ^,^ ^9^ ^]^

Many of the incidences of cancer are associated with increase of these exogenous agents in the human adipose tissue and biological fluids.^[^ ^10^ ^–^ ^11^ ^,^ ^12^ ^]^ Bisphenol A (BPA) also known as 2,2‐bis‐4‐hydroxyphenyl propane, an organic compound that is widely used in the plastic industry to manufacture epoxy resins and polycarbonate has been presented as one of the most harmful EDCs due to its interference with hormone function, mimicking estrogen, and its binding ability to estrogen receptors, which cause an alteration in endogenous hormone synthesis, hormone metabolism, and hormone concentration in the blood.^[^ ^13^ ^,^ ^14^ ^]^ As a results of the harmful effects of BPA, it is necessary to develop methods for BPA determination in the environment.

At present, different methods have been developed for the determination of BPA, such as liquid chromatography‐mass spectrometry,^[^ ^15^ ^]^ enzyme‐linked immunosorbent assay (ELIZA),^[^ ^16^ ^]^ fluorescent analysis,^[^ ^17^ ^]^ molecular imprinting technique,^[^ ^18^ ^]^ flow injection chemiluminescence,^[^ ^19^ ^]^ impedimetric immunosensor,^[^ ^20^ ^]^ capacitor,^[^ ^21^ ^]^ quartz crystal micro‐balance,^[^ ^22^ ^]^ and electrochemical sensor. Among these methods, electrochemical method has been widely explored due to its low cost, fast response, cheap instrument, ease of preparation, and high sensitivity.

Metal‐organic frameworks (MOFs) are widely used as sensor,^[^ ^23^ ^,^ ^24^ ^]^ adsorbents, and catalysis^[^ ^25^ ^–^ ^26^ ^,^ ^27^ ^]^ applications for decades. Their porosities, tunable pore size, and large specific surface area are some of the characteristics that make them useful for these applications. The presence of metal ions or clusters, linked to a mono or multidentate organic molecules makes them versatile compounds.^[^ ^28^ ^,^ ^29^ ^]^ Yingpan et al., used a bimetallic CoNi‐based MOFs for labeling free impedimetric sensor for deoxynivalenol (DON) and salbutamol (SAl).^[^ ^30^ ^].^ Tahir et al.*,* reported the use of luminescence MOFs as potential sensor for various environmental pollutants such as ionic species, volatile organic compounds, and explosives.^[^ ^31^ ^]^ Jiayan et al., synthesized turn‐on MOF‐based luminescent sensor for selective detection of glutathione.^[^ ^32^ ^]^

Owing to their optical, magnetic, electronic, catalytic, and sensing properties, quantum dots (QDs) have been used for different applications such as solar cells, biological imaging, catalysis, and sensors.^[^ ^33^ ^–^ ^34^ ^,^ ^35^ ^]^ The synthesis of QDs in organic solvents produces materials with excellent structural properties. However, due to their high level of hydrophobicity, lack of aqueous solubility, which are important parameters for sensing application, it is necessary to adopt a direct water‐soluble approach for the synthesis of these compounds to reduce their toxicity for practical sensing applications.^[^ ^36^ ^–^ ^37^ ^,^ ^38^ ^,^ ^39^ ^]^ Binary QDs of group II–VI compounds semiconductor such as CdTe and CdSe have emerged as the conventional QDs’ due to their luminescence properties. However, due to the presence of toxic cadmium atom, recent studies have focused on the development of non‐cadmium QDs, such as ternary QDs from group I‐II‐VI elements, such as CuInSe_2_ and AgInSe_2_, which possessed good luminescence properties in comparison to binary QDs.^[^ ^40^ ^–^ ^41^ ^,^ ^42^ ^]^ In addition, they exhibit large full width at half maximum (FWHM) in their photoluminescence spectra and large stoke shift due to their intrinsic defects.^[^ ^42^ ^]^

Various MOFs have been used in the determination of BPA, which revealed that MOFs are good sensing capabilities. Dihui et al., synthesized a bimetallic Ce‐Zn‐MOFs for the determination and sensing of BPA. The bimetallic MOFs was very effective for the detection of BPA with very low detection limit over a wide range of concentrations.^[^ ^43^ ^]^ Haosen et al., studied the selectivity and sensitivity determination of bisphenols compounds in a fluorescent Ga‐MOFs. The sensor not only exhibited good sensing capability with the limit of detection as low as 26.36 μM but possess excellent selectivity toward bisphenols on the synchronous fluorescent changes of turn‐on at 320 nm and turn off at 382 nm^[^ ^44^ ^]^ but little studies have been carried out on the use of ternary QDs as sensor for the detection of BPA.

Driven by the desire to explore the efficiency of ternary QDs conjugated with MOFs for sensing applications, this study focus on the synthesis of MOFs of carboxylic ligands containing amine functional group and, ternary QDs of group I–III‐VI elements and their conjugate as efficient sensors for the detection and sensing of BPA using electrochemical method of detection and compared the efficiency of the conjugate to that of MOFs and ternary QDs separately.

## 2 Experimental Section

### 2.1 Chemicals and Solvents

Copper(II) chloride dihydrate (CuCl_2_)^.^·2H_2_O, 2‐amioterephthalic acid, benzene‐1,3,5‐tricarboxylic acid, indium chloride (InCl_3_), gelatin, glutathione (GSH), dimethylformamide (DMF), thioglycolic acid (TGA), selenium standard solution in nitric acid (SeO_2_ in HNO_3_), ethanol, BPA, potassium ferricyanide [K_3_(Fe(CN)_6_)]. All these chemicals and reagents were purchased from Sigma Aldrich and used as obtained without further purification. All other chemicals and reagents were of analytical grade. Distilled water, deionized water, ultrapure water, were collected from synthetic laboratory in the department of chemistry University of Kwazulu‐Natal.

### 2.2 Apparatus and Measurements

Transmission electron microscopy (TEM) images were obtained from JEOL JEM‐2100 electron microscope (Akishima, Tokyo, Japan). The surface morphology was taken by a scanning electron microscopy (SEM) ZEISS FEGSEM ultra plus (Oberkochen, Germany) at a rating voltage of 15–20 kV at different magnifications as indicated on the SEM images. Fourier transform infrared spectroscopy spectra (FTIR) were recorded on a Bruker Alpha FTIR spectrometer equipped with an Attenuated Total Reflection (ATR) platinum diamond. Melting points were recorded using a Stuart SMP3 melting point apparatus. UV–Vis spectra were recorded on a Perkin‐Elmer Lambda 25 UV–Vis spectrophotometer using dimethylsulfoxide (DMSO) at room temperature and the photoluminescence (PL) study was recorded with Perkin‐Elmer LS 45 fluorescence spectrometer excited between 300 and 500 nm using DMSO at room temperature. All electrochemical studies including differential pulse voltammetry (DPV) cyclic voltammetry (CV) electrochemical impedance spectroscopy (EIS) were carried out in an electrochemical workstation Autolab PGSTAT 302N containing an electrochemical impedance module (EIS). A standard three electrodes system containing a gold or platinum working electrode, silver/silver chloride (Ag/AgCl) wire as a pseudo reference electrode and platinum (Pt) counter electrode were used for the electrochemical experiments at ambient temperatures.

### 2.3 Synthesis of MOFs [Cu_2_(2‐ATA)_2_(1,3,5‐BTA)_2_(H_2_O)_2_] Compound 1

**Scheme 1:**
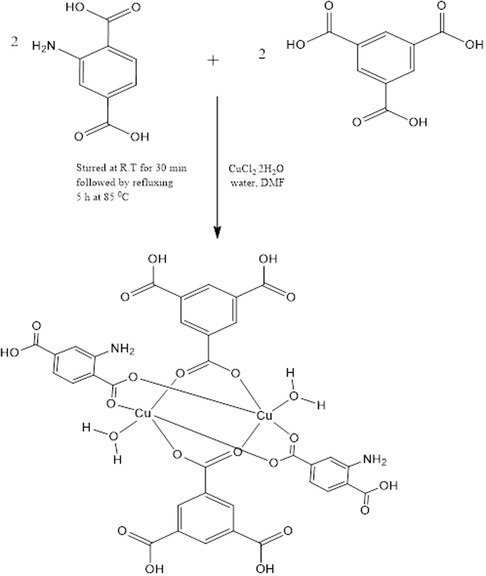
Propose synthesis of MOFs [Cu_2_(2‐ATA)_2_(1,3,5‐BTA)_2_(H_2_O)_2_] compound 1

The synthetic method used by Yang et al.,^[^ ^45^ ^]^ was adopted with some modifications. Copper(II) chloride dihydrate (CuCl_2_)^.^·2H_2_O (2 mmol, 0.340 g) was dissolved in 10 mL of N, N‐ dimethylformamide (DMF) and stirred at room temperature for 30 min, 2‐aminoterephthalic acid (2‐ATA) (2 mmol, 0.362 g) and 1,3,5‐benzene tricarboxylic acid (1,3,5‐BTA) (2 mmol, 0.420 g) was dissolved in 10 mL DMF/water ratio 2:1 each and, both ligands were mixed and stirred at room temperature for 45 min. A green precipitate was obtained when the solution of the metal salt and the ligands were mixed, the whole mixture was then stirred at room temperature for 30 min and later refluxed at 85 °C for 5 h. After 5 h of refluxing reaction, the mixture was precipitated by adding 5 mL of ethanol, centrifuged at 1000 revolutions per minute (rpm) and then filtered, washed with DMF and water in 1:1 ratio. The product obtained was dried at room temperature, kept in oven for 45 min at 50 °C for activation and kept in a desiccator for utilization and further characterization (**Scheme** **1**). Yield: 83%, M.pt: 388 °C. TOF‐MS ESI^+^ (m/z): Anal. Calcd.: 941.67. Found: [M] + 941.91. Anal. Calc. for C_34_H_26_Cu_2_N_2_O_22_ (%): C: 43.37; H: 2.78; N: 2.97; Found (%): C: 42.60; H: 2.97; N: 3.33.

### 2.4 Synthesis of QDs (CuInSe_2_) Compound 2

**Scheme 2:**
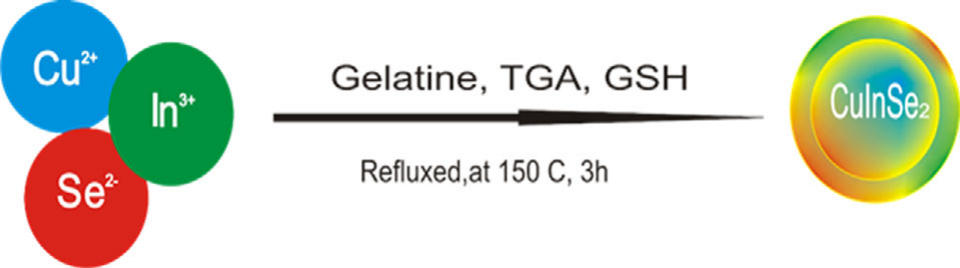
Proposed synthesis of CuInSe_2_.

Hot injection synthetic method used by Latha et al.,^[^ ^46^ ^]^ was used with some modifications (**Scheme** **2**). The synthesis of aqueous soluble gelatin‐stabilized CuInSe_2_ QDs was achieved following this brief method, 1 mL TGA was added to 10 mL water, and the mixture was stirred at room temperature (RT) for 10 min. Then, CuCl_2_·2H_2_O ( 0.34 g, 2 mmol) in 10 mL distilled water, was added to the solution and stirred for 40 min at room temperature. This was followed by the addition of indium chloride (InCl_3_) (0.586 g, 2 mmol) in 8 mL of distilled water. The mixture was refluxed for 1 h at 150 °C. Later, 10 mL SeO_2_ in HNO_3_ solution was then added slowly to the hot solution of CuIn. Sodium borohydride (NaBH_4_) (0.25 g) as a reducing agent for selenium was added to the above mixture, then the whole mixture was refluxed for 3 h at 150 °C. Glutathione (0.309 g, 1 mmol) to reduce the toxicity and prevent the reduction of copper(II) ion was added to the mixture under refluxing and this was followed by the addition of sodium citrate tribasic dihydrate (0.294 g, 1 mmol) for the dispersion of the quantum dots. The whole reaction was performed under inert atmosphere using nitrogen gas. At the end of the reaction time, CuInSe_2_ was precipitated by adding 5 mL of ethanol and centrifuge at 1000 rpm. The ternary QD (CuInSe_2_) was collected, dried, and kept in oven for 30 min at 50 °C for activation.

### 2.5 Synthesis of Composite {CuInSe_2_ @[Cu_2_(2‐ATA)_2_(1,3,5‐BTA)_2_(H_2_O)_2_]} Compound 3

**Scheme 3:**
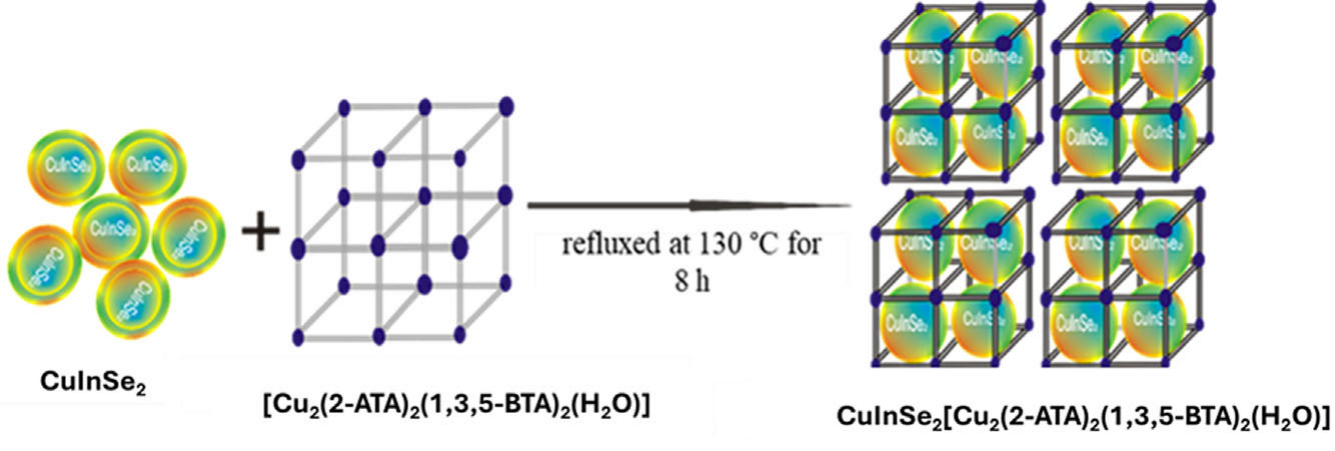
Proposed synthesis of [CuInSe_2_ @[Cu_2_(2‐ATA)_2_(1,3,5‐BTA)_2_(H_2_O)_2_]]

The synthesis of the composite *{CuInSe*_*2*_ *@[Cu*_*2*_ *(2‐ATA)*_*2*_ *(1,3,5‐BTA)*_*2*_ *(H*_*2*_ *O)*_*2*_ *]}* (QDs@MOFs) was achieved by following the ship in the bottle method adopted by Lu Fan et al.,^[^ ^47^ ^]^ with some modifications. A suspension, ultrasonically dispersed of the QDs was prepared by dissolving 25 mg of the QDs in 10 mL distilled water. After the ultrasonication, the dispersed solution was transfer in a 50 mL round bottom flask and stirred. The precursors of the MOFs were dissolved adequately in aforementioned solvents, and the solution of the MOFs was then transferred into the solution of the QDs under vigorous stirring for 15 min. The solutions of the QDs and MOFs were mixed at 1:2 ratio and the whole mixture of the conjugate was then refluxed at 130 °C for 8 hr under nitrogen gas. The resulting QDs@MOFs conjugate was precipitated by adding 5 mL of ethanol, collected by centrifuge at 1000rpm, washed with water and DMF into 1:1 ratio and incubated at 35 °C for 40 min. Compound 3 has a percentage yield of 83% with a melting point of 345 °C. (**Scheme** **3** depicts an example of the conjugate's synthesis).

### 2.6 General Electrode Modification Techniques

The fouling and sluggishness or low response of gold electrode (AuE) are the major's challenges scientist had to overcome to achieve better response in electrochemical studies. Many methods have been employed to modified AuE, which include the simple drop‐dry method to electrodeposition and electropolymerization. The drop‐dry method is governed by adsorption of the substrate onto the surface of the AuE which could result in the chemical interaction between the substrate and to bare AuE through chalcogen element (sulfur/ selenium), while for the electrodeposition and electropolymerization substrates are aligned in a regular pattern via intermolecular interaction or a direct covalent bonding onto the surface of AuE.^[^ ^48^ ^]^ Besides, unconstrained covalent bonding could also occur between the sulfur/selenium containing substrate and the AuE which result into the self‐assembled monolayers (SAMs). In the course of this research simple drop‐dry method was adopted for the modification of the AuE.

## 3 Results and Discussion

### 3.1 FTIR of the Compounds

The spectra of the ligands and compound **1** were compared and assigned, which shows distinct differences. The spectrum of **1,** show a band at 3150 cm^−1^ assigned to υ(N–H) stretch of the amine attached to the 2‐aminoterephthalic acid ligand. The band observed at 3600 cm^−1^ is attributed to υ(O–H) stretch of the uncoordinated hydroxyl group of the carboxylic compound, the υ(O–H) bending vibration of the carboxylic appeared at 1050 cm^−1^. The band observed at 1335 cm^−1^ could be assigned to the υ(C–N) of the aromatic ring interacting with the amine group, which was present in the spectrum of the carboxylic ligand at 1450 cm^−1^. The carbonyl group stretching vibration of the carboxylic group υ(C = O), which was observed at 1760 cm^−1^ in the free ligand shifted to 1600 cm^−1^ upon coordination with Cu(II) ion. The υ(C–H) band of aromatic stretch of the carboxylic group in the free ligand is observed at 3100 cm^−1^, which is also observed at that frequency upon coordination. The bands observed at 1320 and 1640 cm^−1^ which could be attributed to carboxylic stretch υ(C–O) and the stretch of aromatic ring υ(C–C) of the free ligand were shifted to 1250 and1500 cm^−1^ upon coordination. The band observed at 1680 cm^−1^ could be assigned to the stretching υ(C = C) present in the aromatic ring of the ligands. The vibration band of the υ(Cu–O) in the MOFs spectrum is observed at 530 cm^−1^^[^ ^49^ ^]^ (Figure S1 A, Supporting Information).

The spectrum of the GSH show the υ(N–H), υ(O–H), υ(C = O) stretching vibrational bands, and the bending vibration of υ(C–N) at 3400, 3600, 1720, 1360 cm^−1^ respectively, besides, a weak υ(S–H) stretching vibration of the GSH observed at 2550 cm^−1^. The spectrum of the gelatin also shows some stretching vibration bands υ(C–N), υ(O–H), υ(C = O), and υ(N–H) at 2160, 3550, 1730 and 3400 cm^−1^ respectively. The stretching vibration bands for both compounds are also present in the spectrum of QDs (compound **2**) confirming its synthesis. The bands observed at 3400 cm^−1^ could be assigned to υ(N–H) of GSH, at 3550 cm^−1^ could be allotted to υ(O–H) of the gelatin, at 1720 cm^−1^ could be assigned to υ(C = O) of the gelatin, the vibration band observed at 553 cm^−1^ could be attributed to the υ(Cu–In). These observations show that the QDs was successfully synthesized and the surface chemistry of the QDs with hydroxyl, carboxyl, and amine functional groups from the gelatin and GSH are responsible for its water solubility.^[^ ^50^, ^51^, ^52^, ^53^, ^54^ ^]^ (Figure S1 B, Supporting Information).

FTIR spectrum of the composite, QDs@MOFs (compound **3**), which is the conjugate of compounds **1** and **2** reveals major stretching and bending vibrational bands observed in the spectrum of each compound. The major stretching vibrations at the finger‐print region, 553, 530 cm^−1^, could be attributed to υ(Cu–O), υ(Cu–In) observed in the spectra of MOFs and QDs, respectively. The vibrational bands observed at 3200, 2950, 2600, and 3300 cm^−1^, are assignable to υ(N–H), υ(C–H), and υ(S–H), respectively originate from gelatin and glutathione. The stretching vibration band υ(O–H) of the carboxylic group of MOFs has been masked by the amine group present in the QDs. The presence of the major functional groups in the spectrum of the composite, originating from the MOFs and QDs confirm the synthesis of the composite.^[^ ^55^ ^]^ Some other major vibration bands, υ(C–O), υ(C = C), υ(C–N), υ(C = O) at 1320, 1680, 3400, 1720 cm^−1^ respectively observed in the composite were also observed in the spectra of the MOFs and QDs as discussed previously which also confirmed the synergistic interaction of both compounds (Figure S1 C, Supporting Information).

### 3.2 Electronic Spectra, Optical Properties, and X‐ray Diffraction of Compounds 1, 2, 3

The electronic spectrum of compound **1** recorded in dimethylsulfoxide (DMSO) is presented in Figure S2 (A1). The spectrum shows three majors absorption bands in the UV region. The first absorption band observed at 206 nm (48.5436 cm^−1^) is due to the ligand to metal charge transfer (LMCT) transition. The second absorption band at 231 nm (43.290 cm^*−*1^) is assigned to the *π*→π* transition of the benzene ring in the ligand.^[^ ^56^ ^]^ The third absorption band at 345 nm (28.985 cm^−1^) could be due to metal to ligand charge transfer (MLCT) transition back donation between the copper(II) ion and the ligands.^[^ ^57^ ^]^ Intriguingly the *d–d* transition for an octahedral complex was not observed, which confirm the pentagonal geometry around the copper(II) ion.^[^ ^58^ ^,^ ^59^ ^]^ Three major absorptions bands are observed in the spectrum of compound **2** studied in DMSO illustrated in Figure S2 (B1), Supporting Information. All absorption bands are observed in the ultra‐violet region, the absorption at 204 nm (49.019 cm^−1^) could be due to the transition of electrons from n→σ* in the six members ring of gelatin, which is a precursor use in the synthesis of the QD. The other two absorptions bands at 240 nm (41.66 cm^−1^) and 280 nm (35.415 cm^*−*1^) could be attributed to the transition of electrons from n→π* and *π*→π* in thioglycolic acid.^[^ ^60^ ^]^

The absorption bands in the spectrum of compound **3** showed two major absorption bands (Figure S2 (C1, Supporting Information)). The first absorption band at 204 nm (49.019 cm^*−*1^), which was observed in the spectrum of the QDs and the second absorption band observed at 345 nm (28.985  cm^*−*1^), which was due to MLCT observed in the MOFs between. The disappearance of other absorption bands could be due to the interactions between the QDs and MOF through the non‐covalent interaction. The optical bandgap obtained from the ultraviolet‐visible absorbance using ‘Tauc’ relation: (*α*hv)^(1/*γ*)^ versus hv, where “B1”; is the absorption coefficient, “h” is the plan k constant, “v” is the photon's frequency, (1/*γ*) is the electron transition factor, which is ½ in our case for direct bandgap.^[^ ^61^ ^,^ ^62^ ^]^ The energy banggap for compounds **1**,**2**, and **3** are 5.7 eV, 5.2 eV, and 5.6 eV, respectively (Figure S2 (A2), (B2), (C2), Supporting Information). When the valence electrons are excited into the conduction band, the conductivity of a semiconductor increases. The energy bandgap for most semiconductors may vary between 0.1 and 6.2 eV.^[^ ^63^ ^]^ These bandgap energy values revealed that these compounds are semiconductors materials.

There is also an observation of a blue shift in the absorption band edges of the conjugate and MOFs in contrast to the wavelength of the QDs.^[^ ^64^ ^,^ ^65^ ^]^ The diffraction patterns of compounds **1**,**2**, and **3** revealed that major peaks observed in the diffraction patterns of the composite are similar to those observed in the diffraction patterns of MOFs and QDs. The 2*θ* peaks at 15°, 25°, 30° in MOFs which correspond to the lattice plane (331), (421), and (662) of face centred cubic of copper nanoparticles,^[^ ^66^ ^]^ and the 2*θ* peaks at 7° in QDs which correspond to the lattice plane (001) in cadmium selenide in QDs are also observed in the diffraction patterns of the composite. The presence of similar 2*θ* peaks at 7°, 15°, and 25°, which correspond to (001), (331), and (421) lattice plane in the composite confirms the presence of MOFs and QDs in the composite suggesting a mixed phase with the sharpness of peaks suggesting the crystalline nature of the composite (Figure S3, Supporting Information).

### 3.3 Morphology Study of Compounds 1,2,3

**Figure 1:**
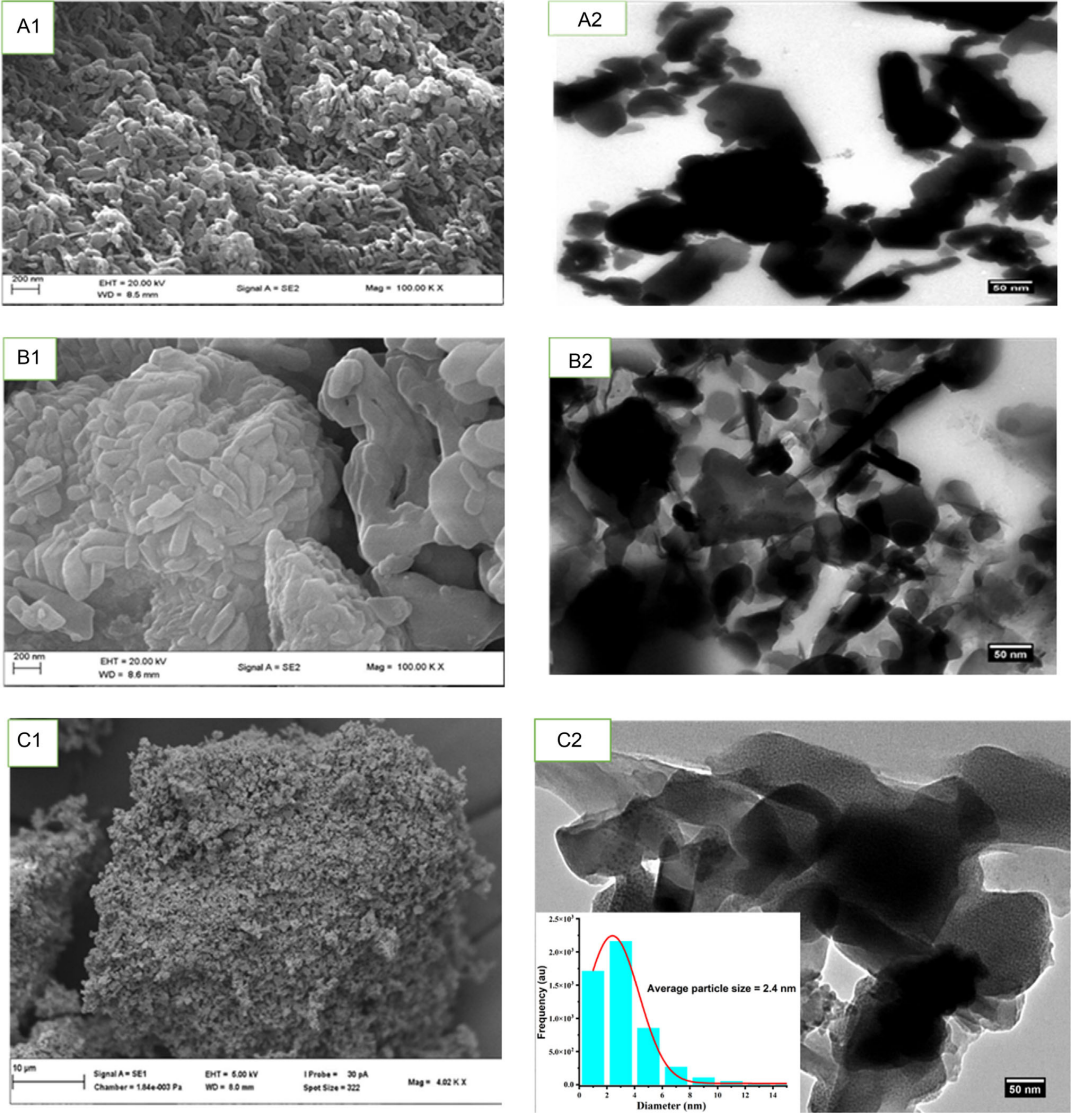
SEM micrographs of compounds **1**,**2**,**3** respectively, represented by **A1**,**B1**,**C1)** and TEM images of compounds **1**,**2**,**3** respectively, represented by **A2**,**B2**,**C2)**, insert is the particle size distribution of compound **3.**

The surface morphology, particle size distributions, crystalline structure, defect, and atomic levels properties of compounds **1**,**2**,**3** were determined by scanning electron micrsocopy (SEM) and transmission electron microscopy (TEM) (**Figure** **1**A–C). SEM micrograph of compound 1 (Figure 3 (A1)) reveals a uniform degree of polydispersity with a spherically shaped and agglomerated MOFs’ particles. The micrograph shows some porosities at the surface of the compound which is a major characteristic of MOFs. The TEM image (Figure 3 (A2)) shows various sizes of compound **1** with well‐defined shapes in several nanometers. The darker color on the image is due to the higher density of atoms, which hinder the incident of the electrons that agrees with the agglomeration of the particles.^[^ ^67^ ^]^ Figure 3 (B1) presents the SEM micrograph of QDs with a spherical and thread‐like plates morphology with smooth surface and some hollow network structure. The TEM micrograph shows agglomerated particles in different sizes and some hollow spaces (Figure 3 (B2)).^[^ ^68^ ^]^ The surface properties of compound **3,** as shown in the SEM micrograph (Figure 3 (C1)), shows fine particles adsorbed to the pores of the MOFs. As the compound was collected after centrifugation, washed with DMF and water in the ratio 1:1 and calcinated in an oven at 35 °C for 40 min. The SEM micrograph shows a possibility of the encapsulation of the QDs into the pores of the MOFs, which are suitable for sensing application. TEM image (Figure 3 (C2)) revealed aggregated irregular shapes with numbers of micropores and crevices of various sizes at the surface.^[^ ^69^ ^]^ The surface properties of these compounds suggest that that they could be suitable materials for electrochemical sensors for BPA determination.^[^ ^68^ ^]^ The average particle size of the composite was calculated and found to be 2.4 nm, which confirm the nanoparticle size of the compound. (Insert Figure 3 C2).

### 3.4 Mass Spectrometry of Compound 1 ([Cu_2_(2‐ATA)_2_(1,3,5‐BTA)_2_(H_2_O)_2_])

The mass spectrum of compound **1** is presented in Figure S4, Supporting Information, the parent ion molecule [M]^+^ is observed at 941.91 m/z ratio in comparison to the calculated molecular weight of 941.76 g.moL^−1^. The peak at 730.34 m/z ratio corresponds to the charged molecule after the fragmentation of one molecule of benzene‐1,3,5‐tricarboxylic acid from the parent ions [M‐(1,3,5‐BTA)]^+^. The picked observed at 943.9 m/z ratio could be attributed to the parent ion with two isotopes of nitrogen (^15^N). The observed peak at 562.15 m/z ratio corresponds to the charged molecule

obtained after a fragmentation of two molecules of 2‐amino‐terephthalic acid and a water molecule [M‐{(2‐ATA)_2_ +(H_2_O)}]^+^. The peak at 646.24 m/z ratio, which is the base peak, correspond to the charged molecule obtained after the fragmentation of one copper, one benzene‐1,3,5‐tricarboxylic acid and one water molecule from the parent ion with two isotopes of oxygen (^18^O). The peak at 674.27 m/z ratio corresponds to the charged molecule obtained after that 2‐amino‐terephthalic acid molecule and a copper atom have been fragmented with deuterium atoms (^2^H) from the parent ion. The peak at 277.9 m/z ratio corresponds to the charged molecule of 2‐amino‐terephthalic acid and a copper ion, which have been fragmented from the parent ion with four deuterium atoms [{(2‐ATA) + Cu}]^+^. The peak at 226.13 m/z ratio corresponds to charged molecules of 2‐amino‐terephthalic acid and water fragmented from the parent ion [{(2‐ATA) + (H_2_O)]^+^. The peak observed at 365.1 m/z ratio corresponds to the charged fragment of two molecules of 2‐amino‐terephthalic acid with an isotope of oxygen (^18^O) and a deuterium (^2^H) [(2‐ATA)]^+^.^[^ ^70^ ^,^ ^71^ ^,^ ^72^ ^,^ ^73^ ^]^

### 3.5 Electrochemical Modification of the Electrodes

**Figure 2:**
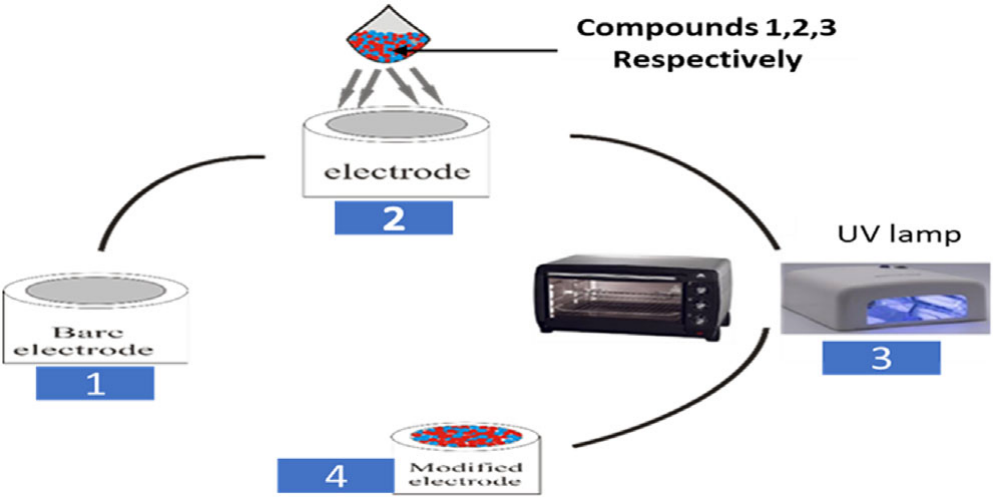
Drop‐dry method of bare electrode modification.

The drop‐dry method is illustrated in **Figure** **2**. The bare gold electrode (AuE) was modified following the drop‐dry (drop‐casting) method of modification. In this method, a drop of the liquid containing a suspension of the particles of interest is first deposited to modify the surface of the bare electrode. An amount of 5 mg of each compound was dissolved in 10 mL ultrapure water and sonicated to obtained a homogeneous suspension. The suspension was casted on the surface of the bare electrode and transfer into a UV lamp chamber for drying under a nitrogen flow to complete the modification.^[^ ^74^ ^]^

#### 3.5.1 Electrochemical Responses of 5 mM Potassium Ferricyanide in 0.1 M KCl at the Surface of the Bare and Modified Electrodes with Compounds 1, 2, and 3 at pH 8

**Figure 3:**
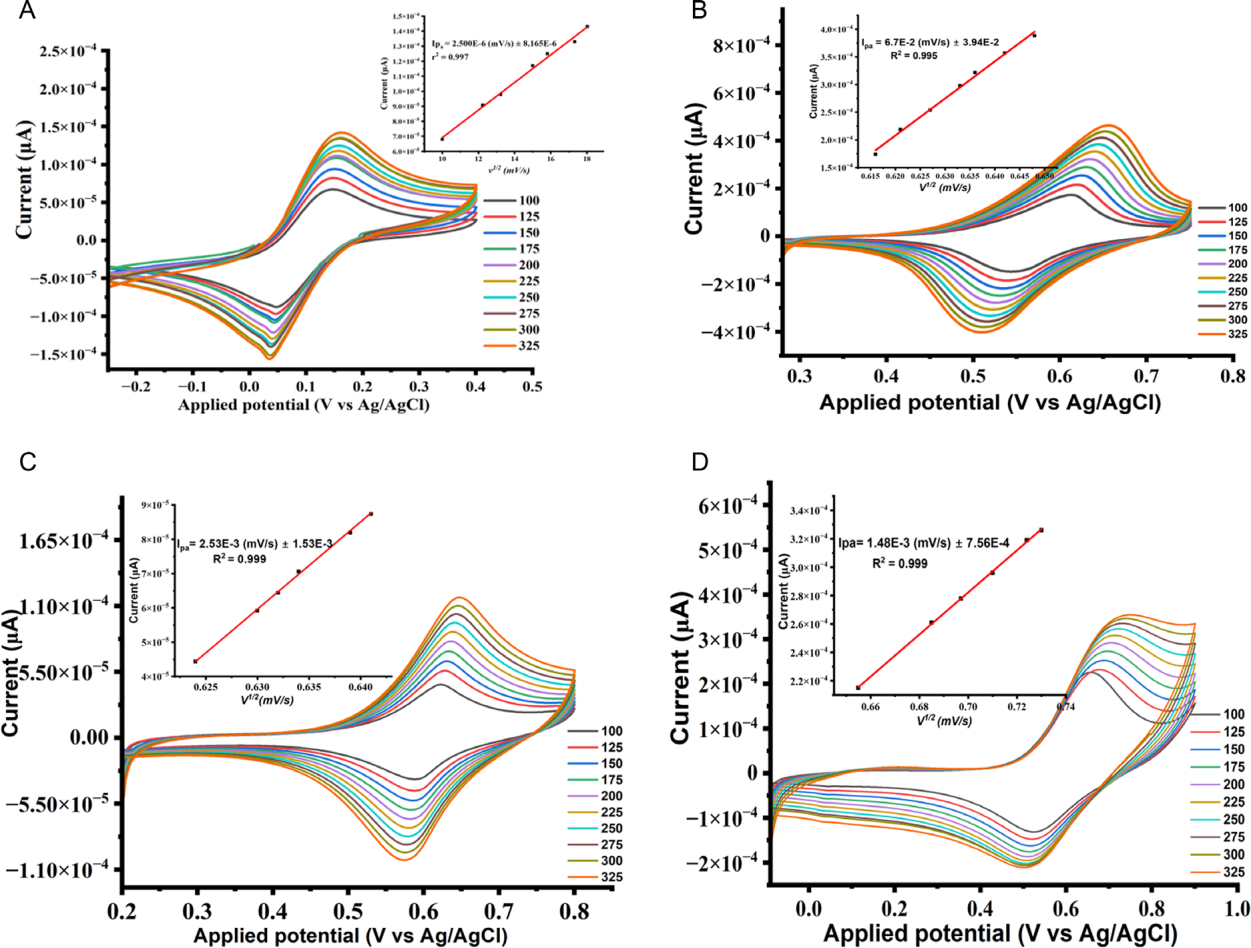
Electrochemical responses of 5 mM potassium ferricyanide [K_3_Fe(CN)_6_] in 0.1 M KCl at the surface of the bare (3A) and modified electrodes with compounds **1**,**2**,**3** (3B, 3C, 3D respectively) at pH 8.

Prior to the analysis of BPA at the surface of the modified electrodes, the charge transfer kinetics properties and the extent of electrons transfer between the surface of the bare, modified electrodes and the electrolyte solution were studied through CV and EIS considering potassium ferricyanide [K_3_Fe(CN)_6_] as exploratory. **Figure** **3** showed the responses of electrodes, and it can be seen that the CV of [K_3_Fe(CN)_6_] at the bare and modified electrodes reveals a pair of a redox peaks. Figure 3A, for the bare electrode, Figure 3B, for the electrode modified with compound **1**, Figure 3C, for the electrode modified with compound **2** and Figure 3D, for the electrode modified with compound **3**. A cyclic sweep window from −0.2 to 1.00 V was considered with an increased in scan rate from 100–300 mV.s^−1^. As the scan rate increases, the reduction peaks current (*I*_pc_) and oxidation peaks current (*I*_pa_) increase linearly with the square root of the scan rate (υ^1/2^), the oxidation peaks current versus the square root of the scan rate ((*I*_pa_) vs (υ^1/2^)) is illustrated in the insert. The linear relationship between the oxidation peak current and the square root of the scan rate indicates that the electrochemical process at the surface of bare and all modified electrodes is controlled by diffusion.^[^ ^75^ ^]^ A summary of redox responses *I*_pa_, *I*_pc_, oxidation peak potential (Epa), reduction peak potential (*E*_pc_) and the peak potential separation (Δ*E*p) at the surface of bare and all the modified electrodes at 100 mV.s^−1^ is presented in **Table** **1**.

**Table 1:** Summary of the redox values and the potential separation at the surface of bare and all the modified electrodes.

Modified electrodes	Oxidation response		Reduction response		Potential separation
	*I*_pa_ [μA]	*E*_pa_ [V]	*I*_pc_ [μA]	*E*_pc_ [V]	Δ*E*_p_ [V] = (*E*_pa_ − *E*_pc_)
Bare	6.745E‐5	0.145	−8.740E‐5	0.048	0.097
MOFs/AuE	1.74 E‐4	0.613	−1.455E‐4	0.549	0.064
QDs/AuE	4.54E‐5	0.622	−3.32E‐5	0.588	0.034
QDs@MOFs/AuE	2.26E‐4	0.655	−1.29E‐4	0.528	0.127

The results presented in Table 1 reveal that the potential separations at the surface of all the modified electrode are less than 300 mV, which indicate that the reversibility of [K_3_Fe(CN)_6_] at these modified electrodes is adequate. In accordance to Nicholson's theory, the standard heterogeneous rate constant (*K°*) increases with decrease in the value of Δ*E*_p_, therefore the modified electrodes facilitate the electrons transfer of [K_3_Fe(CN)_6_].^[^ ^76^ ^]^ Besides, the oxidation peak current of [K_3_Fe(CN)_6_] slightly increases at the modified electrode with the composite when compared to the oxidation peak current at the modified electrodes with compounds **1** and **2**, suggesting that the surface area of the modified electrode with the composite is more favorable and larger for the sensing application of BPA in contrast to the other two modified electrodes. According to Randles–Sevick's Equation ( (Equation 1) ), the oxidation peak current is directly proportional to the surface area of the electrode, the higher the oxidation peak current the higher the surface area of the electrode.

(1) $$I_{\mathrm{pa}}=2.69\times 10^{5}{\mathrm{AD}}^{½}n^{3/2}{\text{ν}}^{1/2}C$$

“*I*_pa_” is the oxidation peak current, “A” is the surface area of the electrode, “D” is the diffusion coefficient, “n” is the number of electrons, “v” is the scan rate, “C” is the concentration of [K_3_Fe(CN)_6_].

#### 3.5.2 EIS

**Figure 4:**
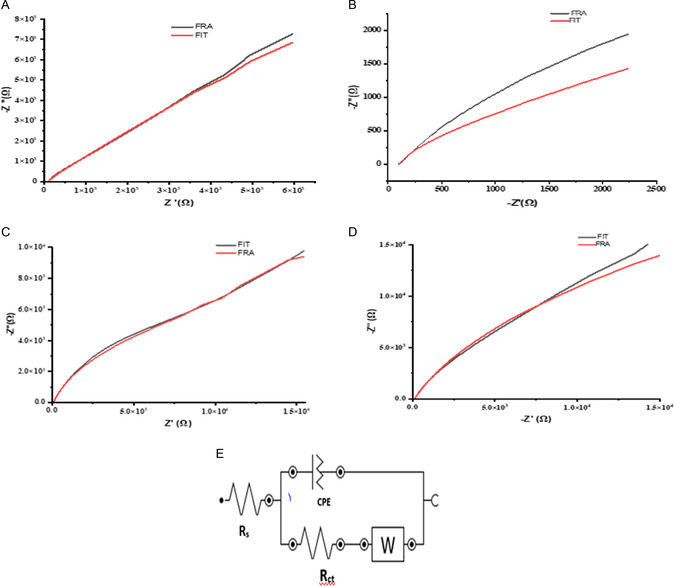
Nyquist plots responses obtained in solution of 5 mM [K_3_Fe(CN)_6_] in 0.1 M KCl prepared in PBS solution pH 8, A) using bare, B) electrode modified with compound **1**, C) electrode modified with compound **2** **,** D) electrode modified with compound **3** **,** E) the equivalent Randles circuit used in the EIS experiments.

**Figure 5:**
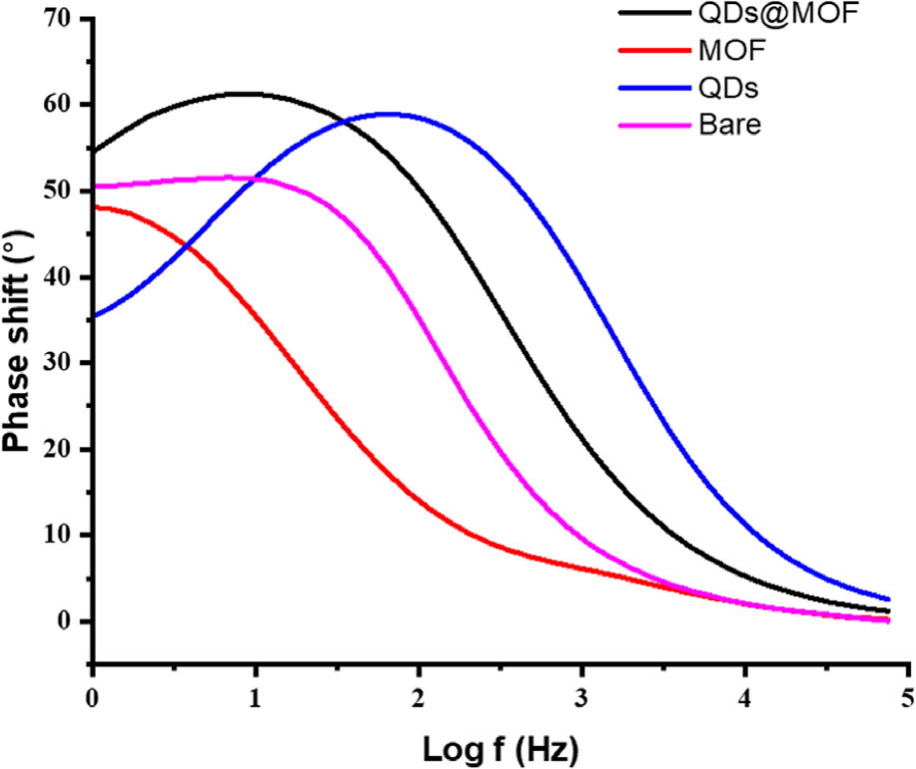
Bode plots obtained with the bare and modified electrodes with MOFs (**1**), QDs (**2**), QDs@MOFs (**3**).

Electrochemical impedance spectroscopy (EIS) is highly effective for examining material characteristics and electrodes responses. The errors structure for impedance measurement, the use of measurement, the process methods and the sensitivity of impedance to the evolution of electrode characteristics are all illustrated through the analysis of impedance data for the reduction of ferricyanide in potassium chloride (KCl) supporting electrolyte.^[^ ^77^ ^]^ The ferricyanide concentration used in this study is 5 mM in 0.1 M of KCl with an applied frequency of 0.1 Hz to 100 KHz. Nyquist plots were used to obtain the impedance across the bare and modified electrodes with compound **1**, **2**, and **3** (**Figure** **4**A–D), respectively**.** The low errors values “n” (*n* < 1) reach between the experimental and fitted data validate the data obtained in this study. In the higher frequency range, the impedance of the electrodes modified with compound **1** illustrates a semi‐circle (curve) while that of the electrode modified with compounds **2** and **3** display almost straight lines. The charge transfer resistance (Rct) that is defined as the diameter of the semi‐circle (curve), which appear at a higher frequency, the electrolyte resistance (Rs), the Warburg impedance (Zw), which is the semi‐circle that appear at a lower frequency, and the constant phase element (CPE) make up the Randle's Sevcik equivalent circuit for the EIS study (Figure 4E **).** In the perspective of semi‐circle (curve), an arc (either high or low frequency) signifies the limit of charge transfer, whilst a diffusion controlled system is identified by a straight line.^[^ ^78^ ^]^ For the given modified electrodes with compounds **1**,**2**, and **3,** the obtained values for Rct were as followed: 40.8 Ω, 9.40 Ω, 4.00 Ω, respectively. These findings imply that, modified electrode with compound **3** has the highest rate of electron transfer due to the lowest Rct value (**Table** **2**). The electrodes’ non‐homogeneity nature has become apparent by the ‘n—values’ (an exponent correlated with the depression angles), which ranges from 0.00 to 0.864, (n < 1).^[^ ^79^ ^]^ The phase shift angles for all the electrodes; bare and modified with compounds **1**, **2**, and **3** illustrated in the bode plots (**Figure** **5**) are as followed: 51°, 48°, 36°, 54°, respectively. These values being less than 90° established that the modification of the electrodes is non‐capacitive.^[^ ^80^ ^]^

**Table 2:** Electrochemical impedance spectroscopy data obtained in 5 mM [K_3_Fe(CN)_6_] in 0.1 KCl at the bare electrode and electrodes modified with compounds 1, 2, 3.

Au electrode	*R*_s_	*R*_ct_	*Z*_w_	CPE[mS]	n
Bare	106 Ω	52.7 Ω	125 μMho	16.6 μMho	0.855
Electrode modified with compound **1**	104 Ω	40.8 Ω	36.5 μMho	10.1 μMho	0.864
Electrode modified with compound **2**	91.2 Ω	9.40 KΩ	60.1 μMho	8.38 μMho	0.763
Electrode modified with compound **3**	90.1 Ω	4.00 Ω	62.2 μMho	7.00 μMho	0.00

“n” the exponential related to the depression angles.

### 3.6 Electrochemical behavior of BPA at the Modified Electrodes with Compounds 1, 2, and 3

**Figure 6:**
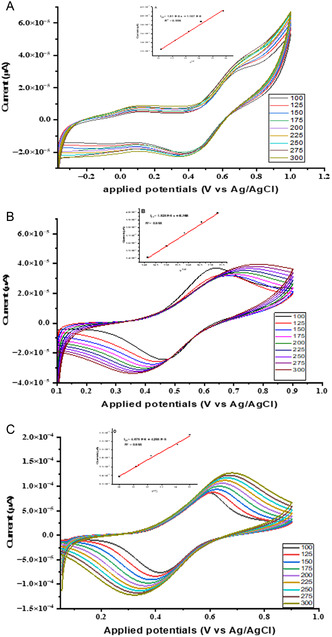
Electrochemical behavior of 3 mM BPA in 0.1 M PBS (pH 8) at the modified electrodes with compound **1** A), **2** B), **3** C) using cyclic voltammetry with an increasing scan rate (100 mV/s–300 mV/s)), insert is the linear increase of the oxidation peak current versus the square root of scan rate.

Electrochemical behavior of 3 mM BPA in 0.1 M PBS at the modified electrodes with compounds **1**, **2**, and **3** at pH 8 were investigated using CV at different scan rate (100–300 mV.s^−1^) (**Figure** **6**A–C). The redox peak currents at the modified electrodes with compounds **1**, **2**, and **3** were observed at the potential window in the range −0.3 V–1.0 V. The redox peaks current witness at the surface of all the modified electrodes suggest that the oxidation of BPA at these electrodes is a complete reaction. From the regression plot, the linear increase in the oxidation peaks current *versus* the square root of the scan rate ((*(I*_pa_ *) vs (υ* ^*1/2*^ *)*) revealed that the electrochemical process of BPA is controlled by diffusion (Figure 6A–C, inserts). A summary of the responses obtained at the surface of all the modified electrodes is presented in **Table** **3**. The highest oxidation peak current (*I*_pa_ = 1.28 E‐4 μA) and the least potential separation (Δ*E*_p_ = 0.356 V) were observed at the surface of the electrode modified with compound **3** (the composite)**.** These values indicate that electrode modified with the composite is much better for the transfer of electrons between the analyte and the modified electrodes surface and the oxidation of BPA is more reversible at the surface of this electrode in contrast to other modified electrodes. Besides, its highest oxidation peak current means that the composite greatly improves the active surface area of the electrode for the sensing application. The electrode modified with compound **3** is therefore more electrochemically active for the sensing of BPA compared to electrodes modified with compounds **1** and **2.** This excellent performance could be attributed to the synergy effect, the conductivity and the intermolecular interaction between MOFs and QDs at the surface of the electrode.^[^ ^81^ ^]^

**Table 3:** Summary of the electrochemical responses for the oxidation peak current (I_pa_) at 100 mV/s scan rate and the separation peak potential (ΔE_p_) at the modified electrodes.

	Electrode modified with MOF	Electrode modified with QDs	Electrode modified with QDs@MOFs
*I*_pa_ at 100 mV/s	3.82 E‐5	4.00 E‐5	1.28 E‐4
*E*_pa_ (V)	0.740	0.785	0.684
*E*_pc_ (V)	0.364	0.352	0.328
Δ*E*_p_ = (*E*_pa_ − *E*_pc_) (V)	0.376	0.433	0.356

#### 3.6.1 Optimization of Experimental Conditions

**Figure 7:**
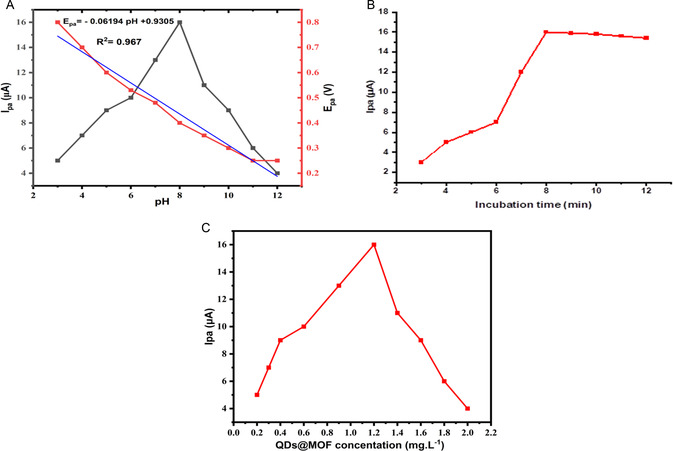
A) Effect of pH of PBS, B) incubation time and C) concentrations of the composite's modified electrode for the electrooxidation nd determination of BPA.

The effect of the pH, concentration of the composite and reaction or incubation time on the electrochemical response of BPA in PBS at the surface of the composite modified electrode were studied to evaluate the optimal conditions for the excellent determination of the analyte. First, the PBS pH optimization was studied, a certain amount of the composite was dissolved in 7 mL ultra‐pure water and studied over a pH range from 2 to 12. Over this range, the oxidation peak current increases gradually as the pH of PBS increases from 2.0 to 8.0, where the oxidation peak current reaches its maximum. This phenomenon may be attributed to the high concentration of protons in solution that can protonate the phenolic ends of BPA molecule causing a good leaving group of water molecule resulting in the increase of more molecules on adsorption site on the composite modified electrode surface. Beyond pH 8.0, the oxidation peak current starts to decrease drastically. The presence of hydroxyl anions may hinder phenolic end reaction from accessing adsorption site on the composite's modified electrode surface. Therefore, PBS at pH 8.0 was selected as the optimum pH for the electrochemical determination of BPA. The linear relationship between the DPV oxidation peak potential (Ep) and the pH was obtained to be as: Ep (V) = −0.06194 pH + 0.9305 (*R* ^2^ = 0.967) (**Figure** **7**A). The absolute value of the slope of −0.06194 V per unit pH is approximately near to 0.059 V per unit pH, suggesting that the number of electrons transfer is equivalent to the number of protons in the electrooxidation and determination of BPA with the electrode modified with the composite (QDs@MOFs/AuE).^[^ ^82^ ^]^

Another crucial variable used to obtain the highest current signal possible for the QDs@MOFs/AuE sensor is the incubation time which was considered between 2 and 12 min. The oxidation peak current was enhanced up to 8 min to reached a plateau and later decreased slightly, suggesting that at 8 min, the surface of the modified electrode was saturated by the adsorbed BPA molecules, which hindered further detection by the modified electrode. Therefore, 8 min was chosen as the optimum incubation time for the electrooxidation of BPA (Figure 7B). The concentration of the composite (QDs@MOFs) suspension plays an important role in the electrooxidation and determination of BPA at the surface of the electrode. In our study, the electrooxidation and determination of BPA was studied by varying the concentration of the conjugate from 0.2 to 2.0 mg.L^−1^ (Figure 7C). The results shows that the oxidation peak current increases with an increase in the concentration of the composite up to 1.2 mg.L^−1^, and a further increase in the concentration resulted in a sharp decrease in the oxidation peak current. The initial increase of the oxidation peak current could be ascribed to the expansion of the conductive electrode surface area and the increase in the accumulation ability of the synergistic effect between the QDs and MOFs, and the decrease in the oxidation peak current with further increase in concentration could be attributed to the limited mass transport of BPA at the surface of the composite modified electrode and the supper saturation of the composite found at the surface of the electrode. Hence a concentration of 1.2 mg.L^−1^ was chosen as the optimum composite concentration for the electrooxidation and determination of BPA.

#### 3.6.2 Electrochemical Determination with Respect to Increase Concentration of BPA Using DPV

**Figure 8:**
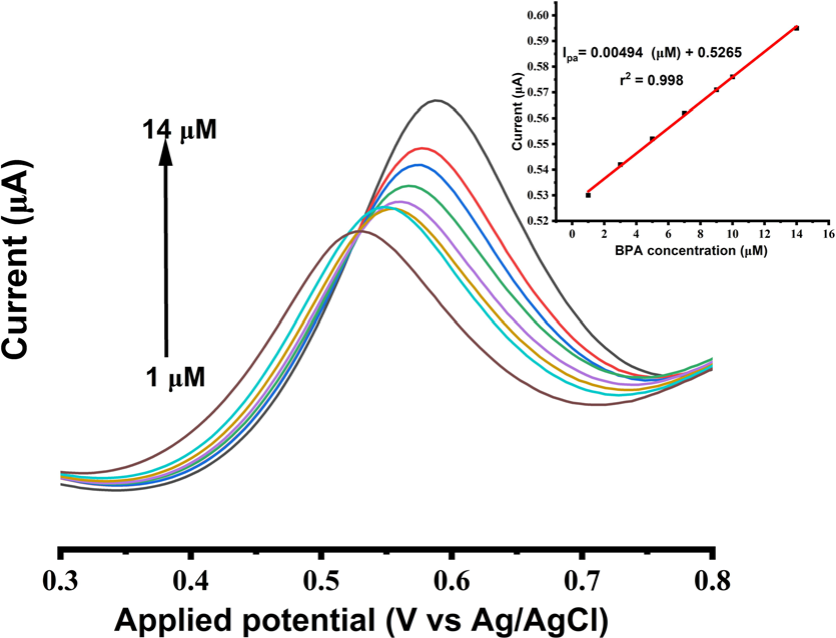
Electrochemical determination of BPA with respect to BPA increase concentration using DPV, insert is the linear regression plot of the oxidation peak current (*I*_pa_) versus BPA concentration.

Differential pulse voltammetry (DPV) with high sensitivity was employed to determine the performance of the electrochemical sensor (electrode modified with compound **3**) (**Figure** **8**). In the insert is the linear regression of the relationship obtained between the oxidation peak current (*I*_pa_) and the various concentrations of BPA. Different concentrations of BPA were prepared, in the range 1–14 μM and the oxidation peak current was evaluated over a potential current in the range from 0.3 to 0.8 V. With an increase in the concentrations of BPA, the oxidation peak current increases linearly as well, which signify that there is an excellent relationship between the concentration of the analyte and the composite modified electrode over this potential voltage range. Several favorable characteristics of composite modified electrode, including its superior electrical conductivity, good sensitivity and catalytic activity could be attributed to its outstanding connection.^[^ ^83^ ^]^ The linear regression equation for the oxidation peak current *I*_pa_ is given as: *I*_pa_ = 0.00494 μM ± 0.5265 with the coefficient of regression *R* ^2^ = 0.998 over the analyte concentration range from 1 to 14 μM. The limit of detection (LOD) and the limit of quantitation (LOQ) were calculated using (Equations 2) & (3).

(2) $$\text{LOD}=3.3\times \left(S^{D}{/}_{m}\right)$$

(3) $$\text{LOQ}=10\times \left(S^{D}{/}_{m}\right)$$

where “SD” is the standard deviation of the oxidation peak current, “m” is the slope of the calibration. The values obtained were 0.470 μM for LOD and 1.425 μM for LOQ (S/N = 3). The shift in the potential current Δ*E*p toward higher value at the increase of BPA concentration indicates that the intercalation of the precursors in the conjugate is efficient. European Union Commission (EUC) established a specific migration limit (SML) of 2.63 E‐6 mol.L^−1^ in 2004.^[^ ^84^ ^]^ The electrochemical sensing technique of BPA, which was achieved using the composite([CuInSe_2_@[Cu_2_(2‐ATA)_2_(1,3,5‐BTA)_2_(H_2_O)_2_]]) at the surface of the gold electrode, with a limit of detection of 0.470 μM meets the EUC standard value for specific migration limit. **Table** **4** displays the results of a comparison between the performance of BPA detection using this composite modified electrode and other previously published results using other compounds as sensors. The LOD result obtained for the detection of BPA in this research work shows a lower value for the limit of detection and a wider detection concentration range, indicating a modest BPA detection at the surface of composite modified electrode.

**Table 4:** Comparison of some parameters of the BPA detection between this developed method and previous published methods.

Sensors	Linear range	Methods employed	Limit of detection	Ref.
PEDOT/GCE	40–410 μM	cyclic voltammetry	22 μM	[86]
glassy carbonelectrode (GCE)	0.02 –20 μM	sensitive electrochemical method	7.5 μM	[87]
NH2‐MIL‐125/RGO/GCE	2 –200 μM	Cyclic voltammetry	0.79 μM	[88]
SWCNT/GCE	10 –100 μM	Cyclic voltammetry	7.3 μM	[89]
cationic surfactant cetyltrimethyl ammonium bromide, Fe_3_O_4_	6.0 × 10 ^−7^ to 1.0 × 10 ^−4^mol L^−1^	Cyclic voltammetry	1.0 × ^10−7 ^mol L^−1^	[90]
Tyrosinase‐MWCN paste electrode	1–16 μM	cyclic voltammetry	1 μM	[91]
MIP/g‐C3N4r/FTO	5 mol . L^−1^ – 100 mol . L^−1^	cyclic voltammetry	1.3 μM	[92]
Copper oxide modified Carbon paste electrode	–	cyclic voltammetry	2.48 μM	[93]
exfoliated graphite (EG)	1.56–50 μM	Square wave voltammetry	0.76 μM	[94]
([CuInSe_2_ @[Cu(2‐ATA)(1,3,5‐BTA)(H_2_O)_2_]]	1–14 μM	cyclic voltammetry	0.46 μM	This work

#### 3.6.3 Electrochemical Determination of BPA on the Modified Electrodes with 1, 2, 3 Using DPV.

**Figure 9:**
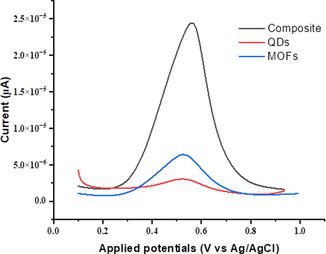
Electrochemical determination of BPA on the modified electrodes with compounds **1**, **2**, and **3** using differential pulse voltammetry (DPV).

The oxidation peak current of the three modified electrodes containing compounds **1**, **2**, and **3** was studied to further investigate the electrochemical determination of BPA using DPV. **Figure** **9** depicts the oxidation peak response obtained at the surface of the modified electrodes using DPV over a window of 0.2 to 1.0 V. The highest value for the oxidation peak current obtained at the electrode modified with the composite was 2.5 E‐5 μA, while the oxidation peak current obtained at the modified electrodes with MOFs and QDs have values ranging between 2.5 E‐6 and 7.5 E‐6 μA, which confirm the excellent performance of the composite modified electrode over the electrodes modified with MOFs and QDs. This outstanding performance could be attributed to the synergistic effect between the MOFs and QDs used to fabricate the composite, and this synergistic effect promotes and facilitates the movement of electrons between the electrolyte and surface of this electrode.

### 3.7 Stability, Reproducibility, and Selectivity

**Figure 10:**
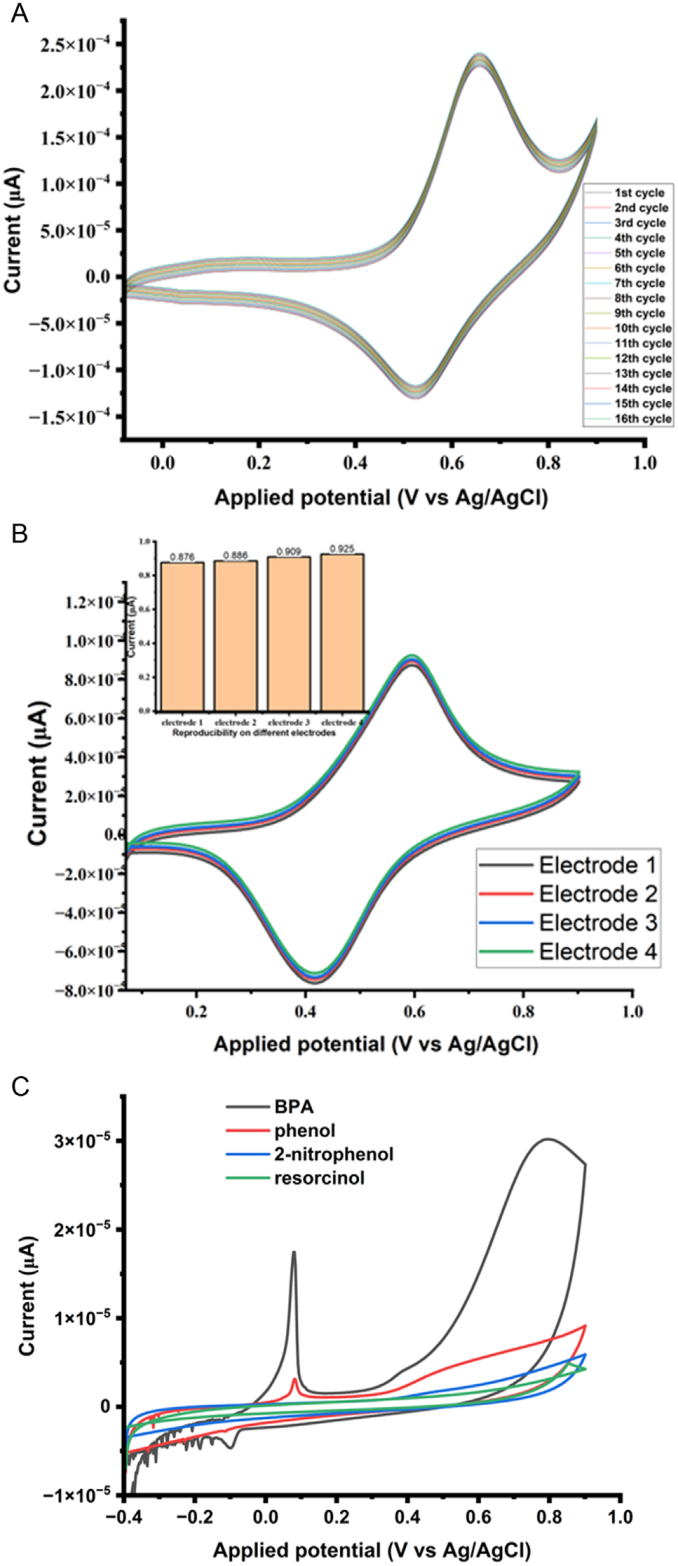
CV's of 5 mM [K_3_Fe(CN)_6_] in 0.1 KCl obtained for 15 cycles on the modified electrode with compound **3:** A) Stability, B) CV's of 5 μM BPA in 0.1 M PBS (pH 8) at four different electrodes modified with compound **3**, insert is the bar chart of response of current versus different electrodes: reproducibility, C) CV responses obtained for BPA and others interfering substances at the surface of composite modified electrode for selectivity.

One of the significant parameters in accessing the electrodes performance is stability of the modified electrode. Accurate detection and reduction in detection errors are only possible with highly stable modified electrodes. Electrode modified with compound **3** was fabricated and evaluated for his stability using 5 mM [K_3_Fe(CN)_6_] in 0.1 M KCl as the test solution at 125 mV.s^−1^. The stability experiment was performed for 15 days with one day interval (**Figure** **10**A). The difference between the oxidation peak current of the first day cycle scan and the last day cycle scan was found to be around 5.26% after all the 15 cycle scans. When the modified electrode is not in use, it is kept in oven at 35 °C. These findings indicate that the stability of the electrode modified with compound **3** is modest.

To ascertain the reproducibility of the modified electrode with compound **3,** four (4) different electrodes were also modified with the same compound. A CV response of 5 μM of BPA in 0.1 M PBS at pH 8 for the four (4) different modified electrodes was studied under optimum conditions (Figure 10B). All the four different modified electrodes exhibited similar electrochemical responses over a scan rate of 125 mV.s^−1^. Insert is the bar chart of the responses of the oxidation peak current of the four different modified electrodes. The relative standard deviation (RSD) for the responses obtained was calculated to be 2.21%, which suggest that the modified electrode with compound **3** is reproducible.

To access the composite modified electrode selectivity, a competitive recognition study was also conducted. The optimum concentration of the composite modified electrode used in this part of this study was the same one employed in the BPA sensing experiment above. The interfering substances considered were those attributed to the phenol classes of compounds: resorcinol, 2‐nitrophenol and phenol. 5 μM BPA in 0.1 M PBS solution (pH 8) was prepared and 10 μM of the interfering substances were also prepared at the same conditions for this experiment. These compounds have the same solubility properties as the preference analyte because of the phenolic functional group present in them all. CV was used to study the selectivity and some current responses were recorded (Figure 10C **)**. Over a potential window range of −0.4–10 V, it could be observed that the current response of the composite modified electrode for BPA determination is the maximum with a 3.0 E‐5 μA with a shoulder peak current response around 1.73E‐5 μA. For the phenol compound a faint current response was obtained around 5.15 E‐6 μA in addition to a minimal shoulder peak current response obtained around 2.84E‐6 μA. The other two interfering substances didn’t have any peak current response at the surface of the composite modified electrode. The result obtained from the cyclic voltammograms reveals that the composite modified electrode is selective to BPA due to its highest values of peak current response, and this could be due to the presence of the alkyl group (electron donating species) present in BPA compound allowing interaction between BPA and the composite at the surface of the electrode.

### 3.8 Photoluminescence Study of the [CuInSe_2_@[Cu_2_(2‐ATA)_2_(1,3,5‐BTA)_2_(H_2_O)_2_]]/AuE with Increase of BPA Concentrations.

**Figure 11:**
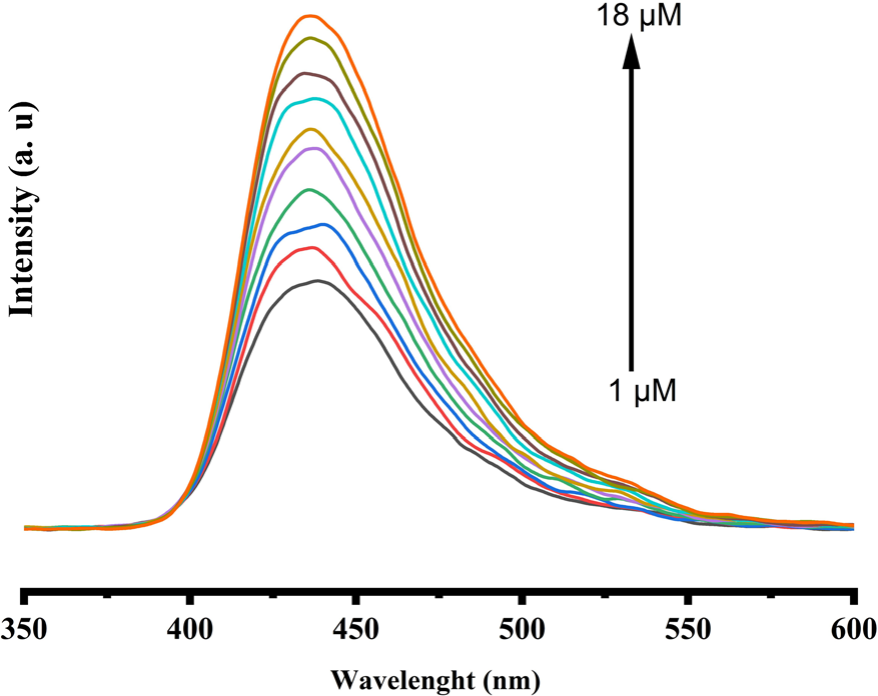
Photoluminescence response of composite with BPA increase in concentration.

The influence of the concentration of BPA (1 to 18 μM) on the photoluminescence of the composite is shown in **Figure** **11**. At the excitation wavelength of 405 nm, a PL emission band at 436 nm is observed for the composite, which could be attributed to the strong intercalation between the MOFs and QDs, and this is due to the chelating and electrostatic interactions between the precursors that made up the composite with efficient electronic transition. The presence of the high number of electrons creates a proportional electronic structure (heterojunction structure), which causes a reduction in bandgap between the valence and conduction band of the composite and facilitate an excitation of electrons and an increase of emission intensity.^[^ ^85^ ^]^ Besides the formation of this electronic structural relationship, synergistic effect between the analyte and the composite, and electron–holes pair are formed on electronic levels of the composite that lead to increase of charge separation, which increases the PL emissions intensities. Therefore, an increase in the concentration of the analyte will cause an increase in the emission intensity of the composite at 436 nm.

### 3.9 Real Water Sample Analysis

Various water samples were analyzed for BPA using the composite modified electrode sensor with the aim to establish its suitability, practicability and potential for real water sample analysis. Tap water (sample 1), river water near a school (sample 2), and flowing stream water at the entrance of college of agriculture Pietermaritzburg, Scottsville, South Africa (sample 3). Standard addition method (SAM) was used to carry out this study using DPV after the water samples were filtered using 0.55 μm filter membrane. Following the spiking recovery (addition 2 mL of 5 mM of BPA stock solution to 10 mL of each of the three water samples), the concentration of BPA in the three water samples were found approximately to be 5.47, 45.23 and 65.82 μM respectively.

3 mL of each water samples were added to 7 mL phosphate buffer solution (PBS) (pH 8), about 2–6 μM of BPA were added to the samples, the results were recorded using DPV (**Table** **5**). This table revealed that percentage recovery for each water sample increases as the added concentration increases. The percentage recovery varies from 96.27 to 100.63% and the relative standard deviation (RSD) considering five (5) parallel reading was in the range of 3.93–5.96%. These responses obtained at the surface of the composite modified electrode showed the practicability of the fabricated sensor for the determination of BPA in real water samples from the environment. The highest value of the relative standard deviation, which is less than 5%, demonstrate that the recovery responses are adequate and reasonable.

**Table 5:** BPA determination in different water samples (n = 5).

Water Samples	BPA Concentration (μM)	Added concentrations [μM]	Found [μM]	% Recovery	RSD [%]
Sample 1	5.47	2	7.34	98.37	3.81
		4	9.33	98.62	3.39
		6	11.54	100.63	4.54
Sample 2	45.23	2	45.95	97.29	4.30
		4	48.29	98.09	4.29
		6	50.32	98.23	4.92
Sample 3	65.82	2	65.29	96.27	5.96
		4	68.66	98.34	4.99
		6	72.25	100.60	4.53

## 4 Conclusion

In this study, electrochemical sensors were fabricated for the determination of BPA using MOF, QDs, and their composite to modify the surface of gold electrode following a drop‐dry technique. The experimental electrochemical responses of fabricated sensors were evaluated using CV and the electrochemical response obtained by the composite modified gold electrode was significantly better with a linear regression plot of the oxidation peak current versus square root of the scan rate and a coefficient of regression R^2^ = 0.995. This excellent performance of the composite modified electrodes could be attributed to the synergistic effect between the MOFs and QDs, their efficient intercalation properties, their excellent electrical conductivity, and the large surface area of the electrode. over a wide range of BPA concentration of 1 to 14 μM, a limit of detection and limit of quantitation were found to be 0.470 and 1.425 μM, respectively. A comparison of these values to other sensors employed in the determination of BPA from the literature proved that the response of the composite modified gold electrodes in this study is better for the determination of BPA. Other experiments were also performed to ascertain the stability, reproducibility and selectivity of the composite modified gold electrode. Analyses of BPA from water samples confirmed the composite modified electrode sensor could be used for practical application.

## Conflicts of Interest

The authors declare no conflicts of interest

## Supporting information

Supplementary Material

## Data Availability

The data that support the findings of this study are available in the supplementary material of this article.
