# Supplementary material for: Electrochemical Determination of Bisphenol a Using a Drop‐Dry Modified Gold Electrode with Metal–Organic Framework, Quantum Dots, and their Composite
Source: ChemistryOpen. 2025 Aug 7;14(12):e202500327. doi: 10.1002/open.202500327 (PMC12680571; doi:10.1002/open.202500327)
Supplement: Supplementary file 1 — Supplementary Material [file OPEN-14-e202500327-s001.pdf]

Electrochemical Determination of Bisphenol A Using a Drop-Dry Modified Gold Electrode with Metal-Organic Framework, Quantum Dots and Their Conjugate

Solomon O. Oloyede and Peter A. Ajibade*

School of chemistry and physics University of Kwazulu-Natal Private Bag X01, Pietermaritzburg 3209, South Africa

***Correspondence** : ajibadep@ukzn.ac.za

Supplementary information

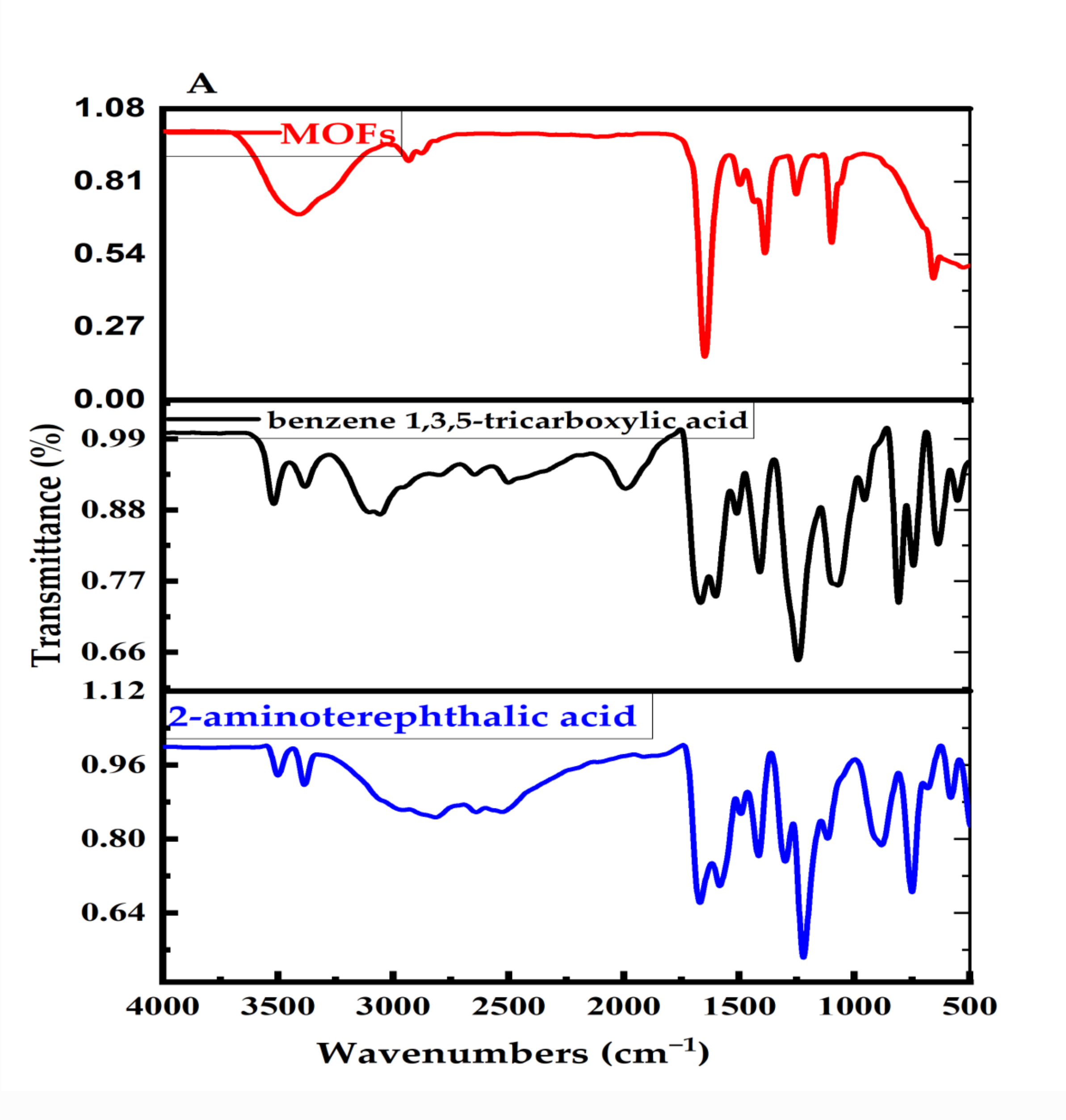

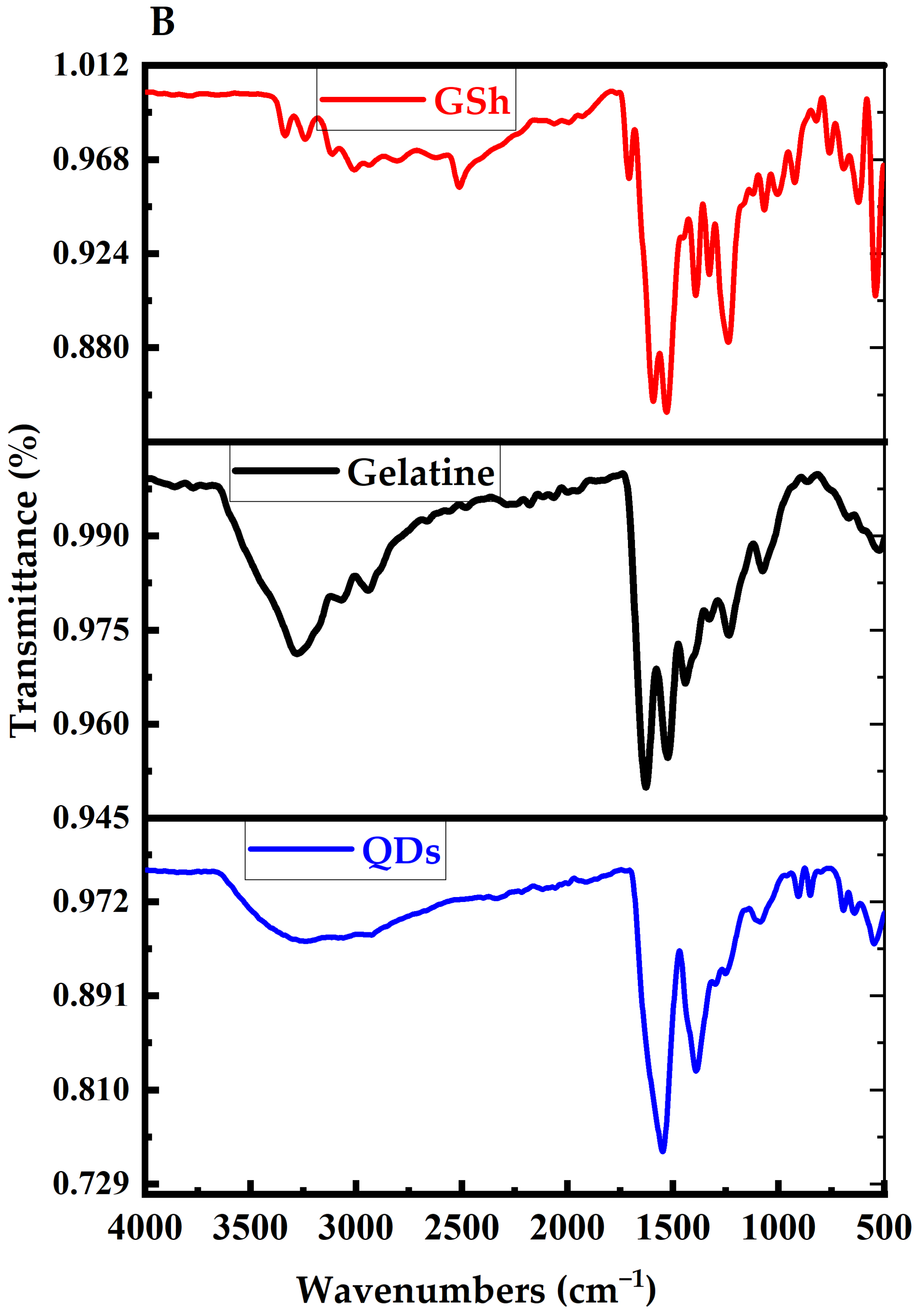
B
GSh
Gelatine
QDs
Transmittance (%)
1.012
0.968
0.924
0.880
0.990
0.975
0.960
0.945
0.972
0.891
0.810
0.729
4000
3500
3000
2500
2000
1500
1000
500
Wavenumbers (cm^{-1})

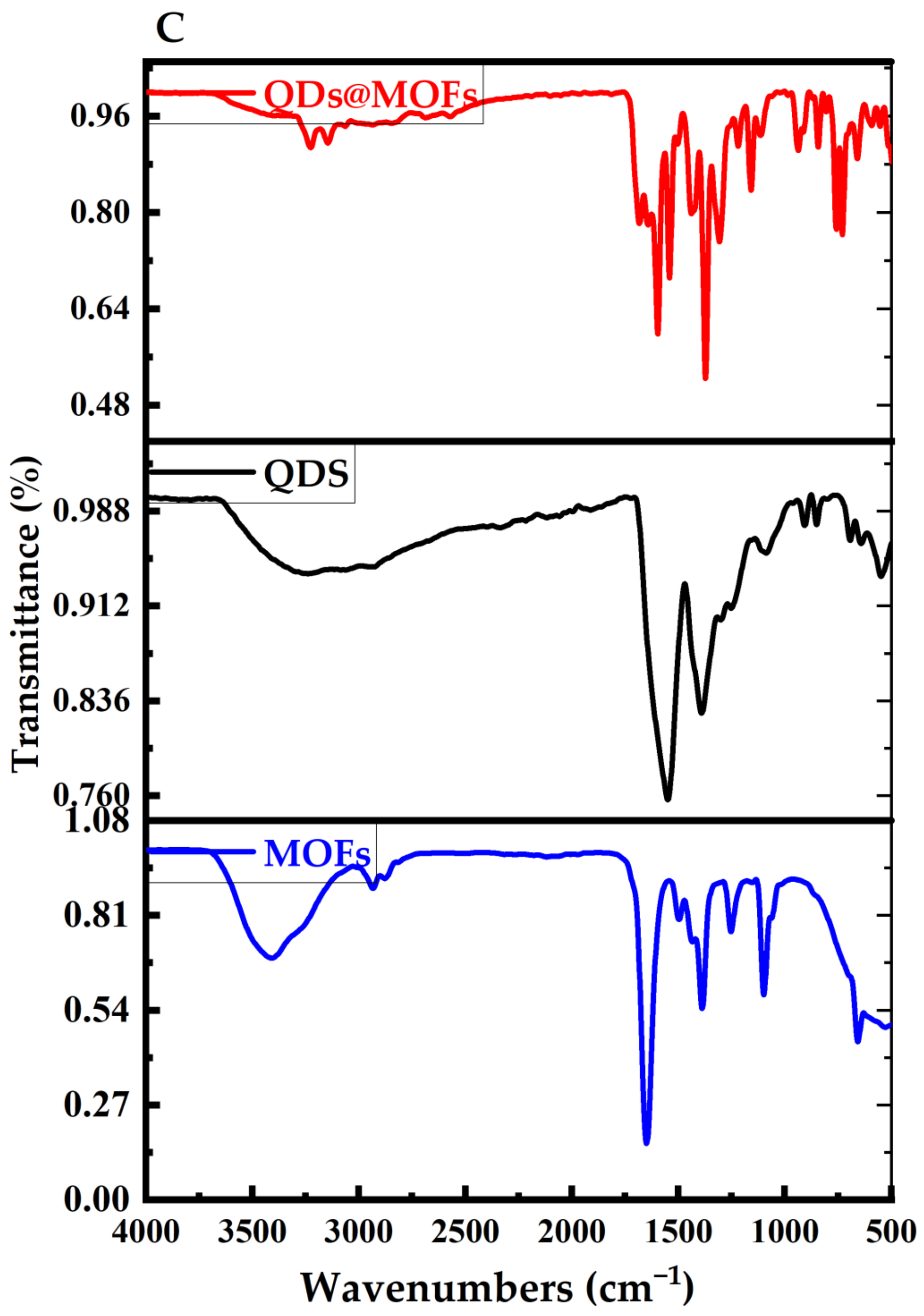

Figure S1: FTIR of compounds **1** (A), **2** (B), **3** (C)

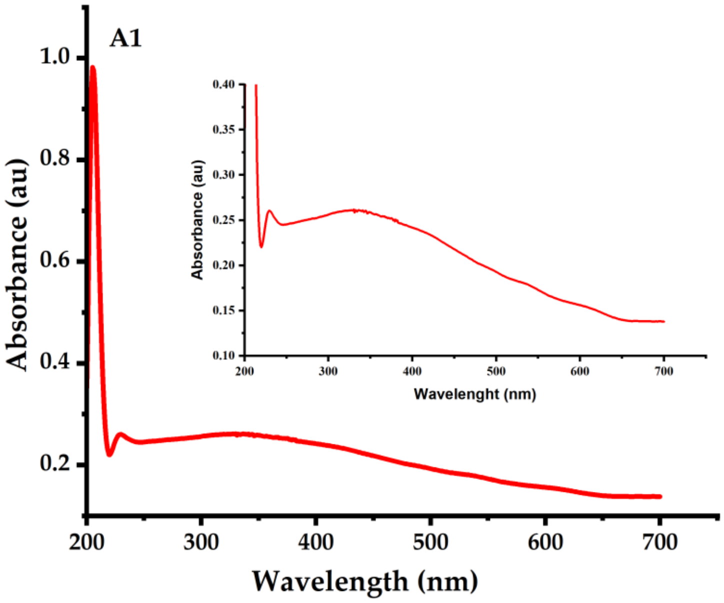
A1
Absorbance (au)
Wavelength (nm)
1.0
0.8
0.6
0.4
0.2
200
300
400
500
600
700
Absorbance (au)
Wavelenght (nm)

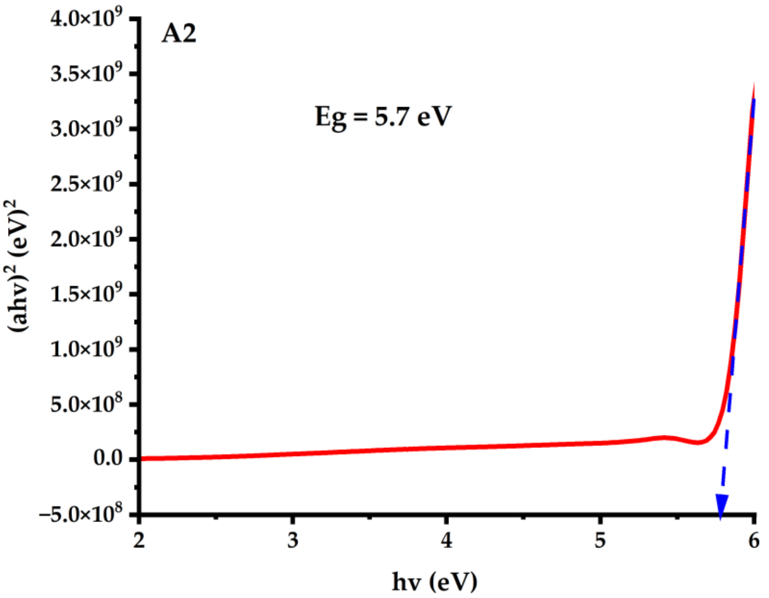
A2
Eg = 5.7 eV
(ahv)2 (eV)2
hv (eV)
4.0×10^9
3.5×10^9
3.0×10^9
2.5×10^9
2.0×10^9
1.5×10^9
1.0×10^9
5.0×10^8
0.0
−5.0×10^8
2
3
4
5
6

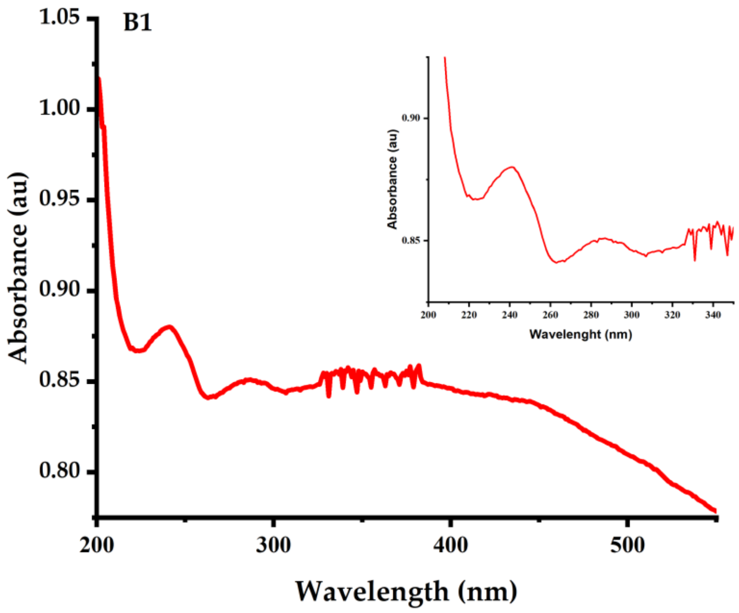
B1
1.05
1.00
0.95
0.90
0.85
0.80
Absorbance (au)
200
300
400
500
Wavelength (nm)
Absorbance (au)
0.90
0.85
200
220
240
260
280
300
320
340
Wavelenght (nm)

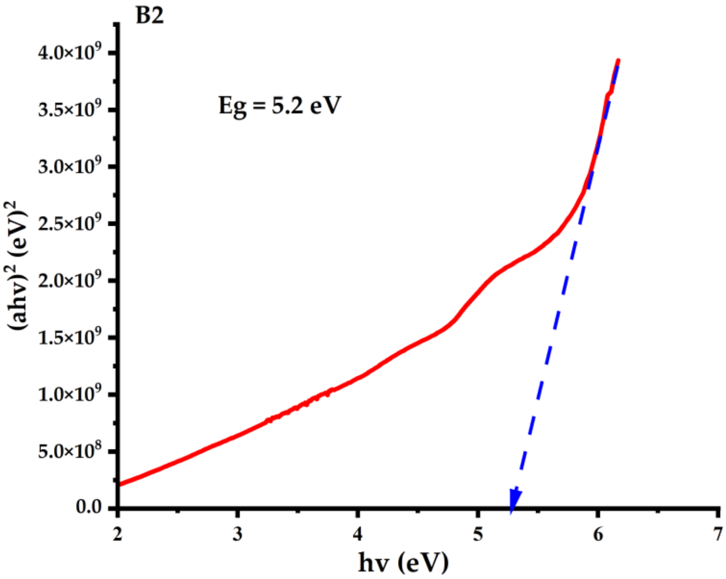
B2
Eg = 5.2 eV
4.0×10⁹
3.5×10⁹
3.0×10⁹
2.5×10⁹
2.0×10⁹
1.5×10⁹
1.0×10⁹
5.0×10⁸
0.0
(ahv)² (eV)²
2
3
4
5
6
7
hv (eV)

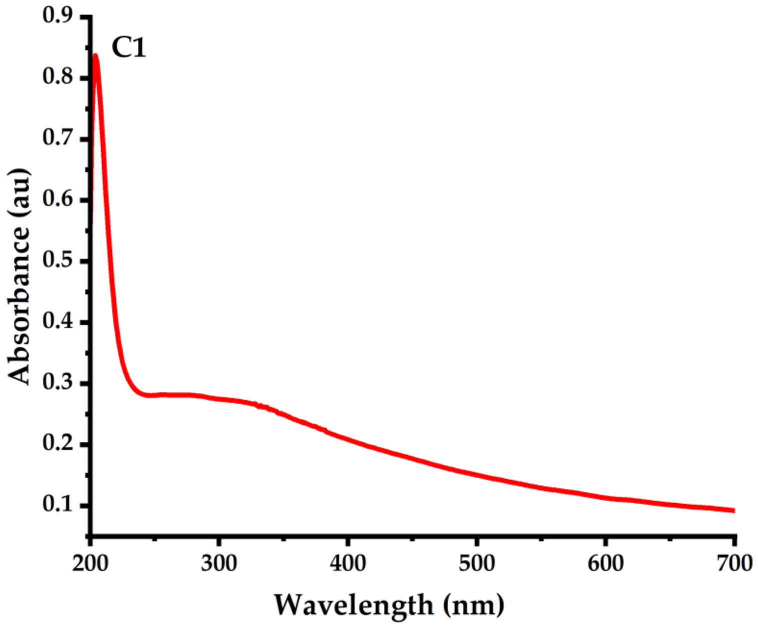

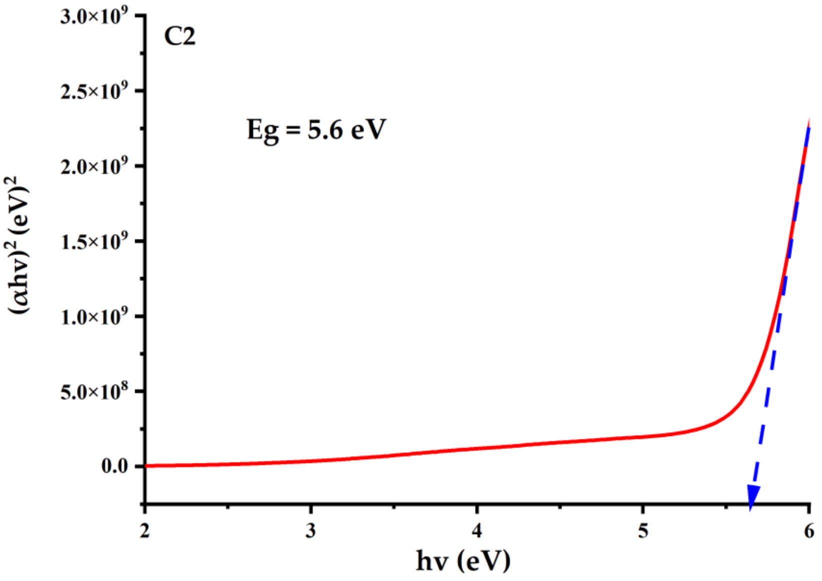

Figure S2: Ultra-violet visible and band gap energy of compound **1** (A1, A2), **2** (B1, B2) **3** (C1, C2) respectively.

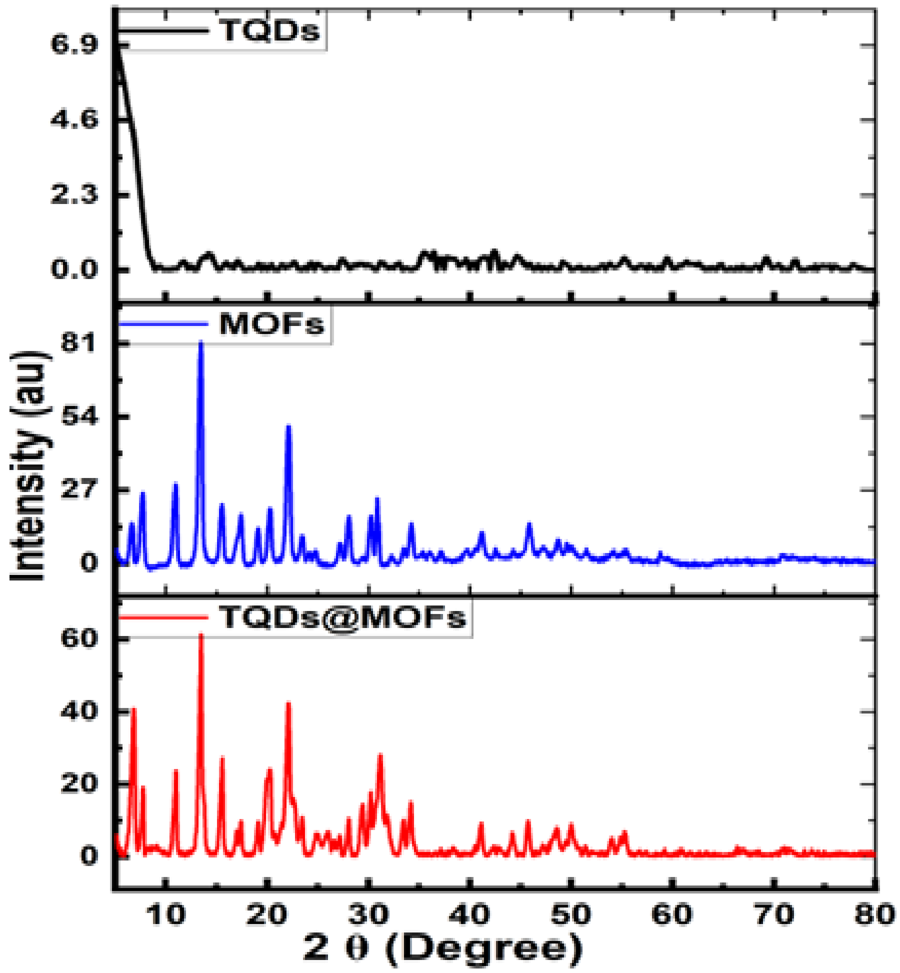

Figure S3: PXRD pattern of compounds (TQDs, MOFs, TQDs@MOFs)

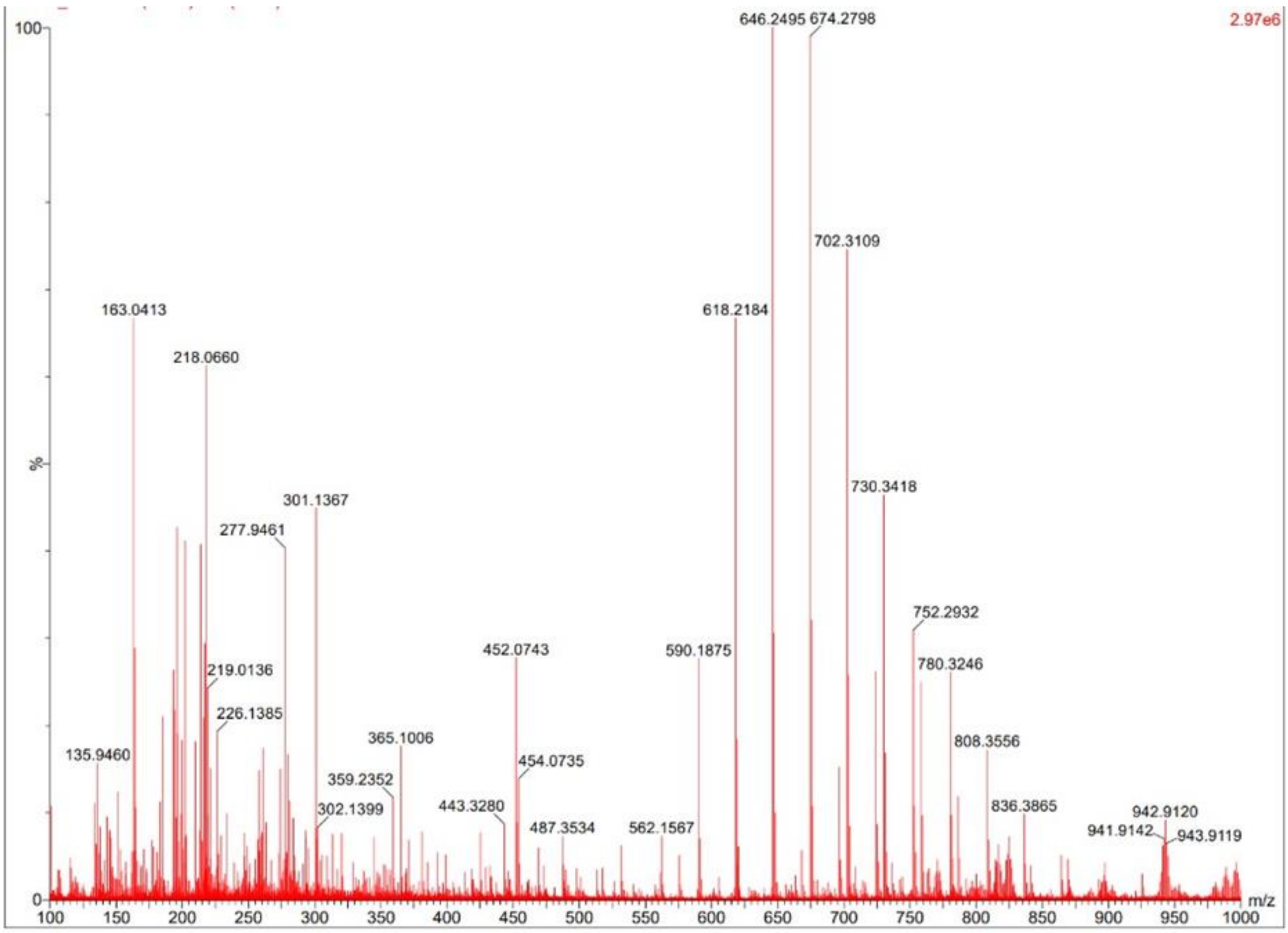

Figure S4: Mass spectra of MOF [Cu_2(2-ATA)$_2$(1,3,5-BTA)$_2$(H_2O)$_2$] **(**compound **1)**
